# Nonamagnesium diruthenium, Mg_9_Ru_2_

**DOI:** 10.1107/S2414314625003050

**Published:** 2025-04-11

**Authors:** Junhui Li, Huizi Liu, Yibo Liu, Bin Wen, Lifeng Zhang, Changzeng Fan

**Affiliations:** a State Key Laboratory of Metastable Materials Science and Technology Yanshan University,Qinhuangdao 066004 People’s Republic of China; b School of Mechanical and Materials Engineering North China University of Technology,Beijing People’s Republic of China; c Hebei Key Lab for Optimizing Metal Product Technology and Performance Yanshan University,Qinhuangdao 066004 People’s Republic of China; Vienna University of Technology, Austria

**Keywords:** crystal structure, high-pressure synthesis, inter­metallics, Mg–Ru system

## Abstract

A monoclinic phase with chemical composition of Mg_9_Ru_2_, nona­magnesium diruthenium, was obtained through high-pressure sinter­ing of a mixture with an initial chemical composition of MgRuB. The Mg_9_Ru_2_ phase crystallizes in the *C*2*/c* space group with *Z* = 8 and is isotypic with the previously reported In_0.74_Ir_3.3_Mg_17.96_ and In_1.9_Ir_3_Mg_17.1_ phases.

## Abstract 2

A monoclinic ordered Mg_9_Ru_2_ phase with *C*2*/c* space-group symmetry rather than previously reported cubic Mg_44_Ru_7_ or Mg_3_Ru_2_ was obtained *via* high-pressure sinter­ing during the effort of synthesizing a target Mg—Ru—B compound.

## Graphical abstract

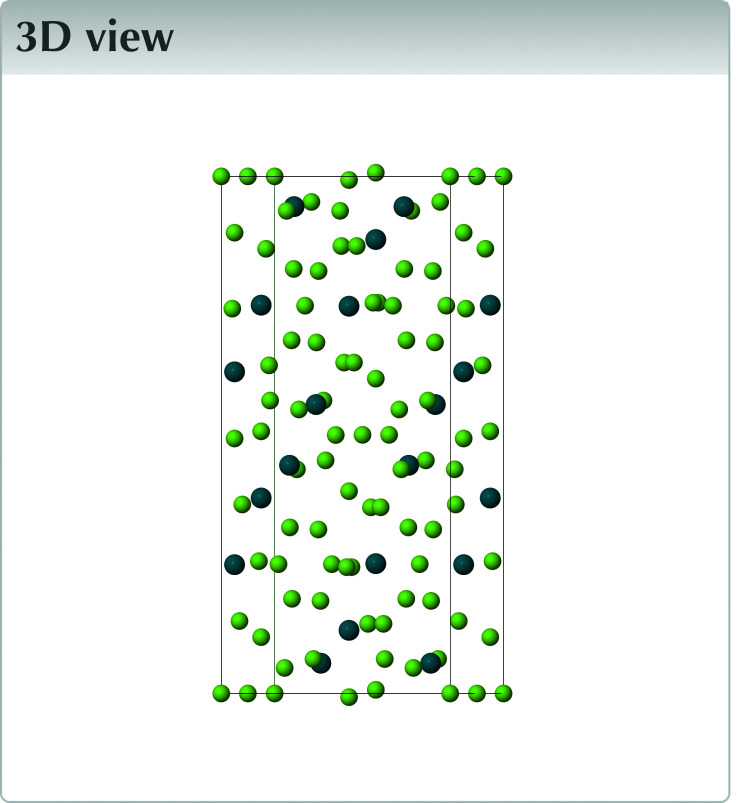

## Structure description

In the B–Mg–Ru system, the potential existence of deca­gonal quasicrystals has been validated through first-principles calculations. These phases comprise a large amount of the non-metallic element boron and do not belong to any known quasicrystals. Schweitzer & Jung (1985 ▸) identified ortho­rhom­bic B_4_Mg_2_Ru_5_ and B_11_Mg_5_Ru_13_ as approximant phases to assumed deca­gonal quasicrystals. Miyazaki *et al.* (2007 ▸) conducted further experimental studies on B–Mg–Ru compounds synthesized at 1673 K, revealing four novel approximant phases on basis of electron diffraction patterns. It should be noted that only the lattice parameters of the four phases have been given in the aforementioned studies while crystal structure models have not been provided.

In a current study aimed at the preparation of a phase with composition MgRuB, crystals of Mg_9_Ru_2_ were obtained serendipitously. The Mg–Ru binary system remains relatively underexplored, and only two phases have been reported up to now besides the new phase Mg_9_Ru_2_: Westin & Edshammar (1973 ▸) investigated the cubic inter­metallic compound Mg_3_Ru_2_ with a *β*-manganese type of structure and Mg_43.83_Ru_7.17_ adopting the Ir_7_Mg_44_ type of structure. They employed Guinier–Hägg and Weissenberg techniques to study X-ray powder and single-crystal data. Pöttgen *et al.* (2008 ▸) further refined the structure of Mg_3_Ru_2_ and conducted a detailed analysis of the chemical bonding.

The lattice parameters of Mg_9_Ru_2_ (Table 1 ▸) are similar to two phases in the In–Ir–Mg system. Hlukhyy & Pöttgen (2005 ▸) synthesized In_0.74_Ir_3.3_Mg_17.96_ and In_1.9_Ir_3_Mg_17.1_ in the Mg-rich part of the In–Ir–Mg system, both of which belong to the space group *C*2*/c*. The lattice parameters for In_1.9_Ir_3_Mg_17.1_ are *a* = 9.8339 (8), *b* = 22.114 (2), *c* = 8.4955 (7) Å, *β* = 105.757 (6)°. Its asymmetric unit contains two Ir sites, two Mg sites with full occupation and nine mixed-occupied (Mg/In) sites with concentrations ranging between 1.2 and 14.8 at.% for In. The authors proposed the possible existence of a ternary compound with the ideal composition InIr_3_Mg_18_ and subsequently synthesized a compound with a near-ideal composition, In_0.74_Ir_3.30_Mg_17.96_, with lattice parameters *a* = 9.791 (1), *b* = 21.974 (2), *c* = 8.482 (1) Å, *β* = 105.79 (1)°. As it turned out during crystal structure refinement of Mg_9_Ru_2_, the crystal structure of the binary phase is isotypic with the two In–Ir–Mg phases.

**Figure 1:**
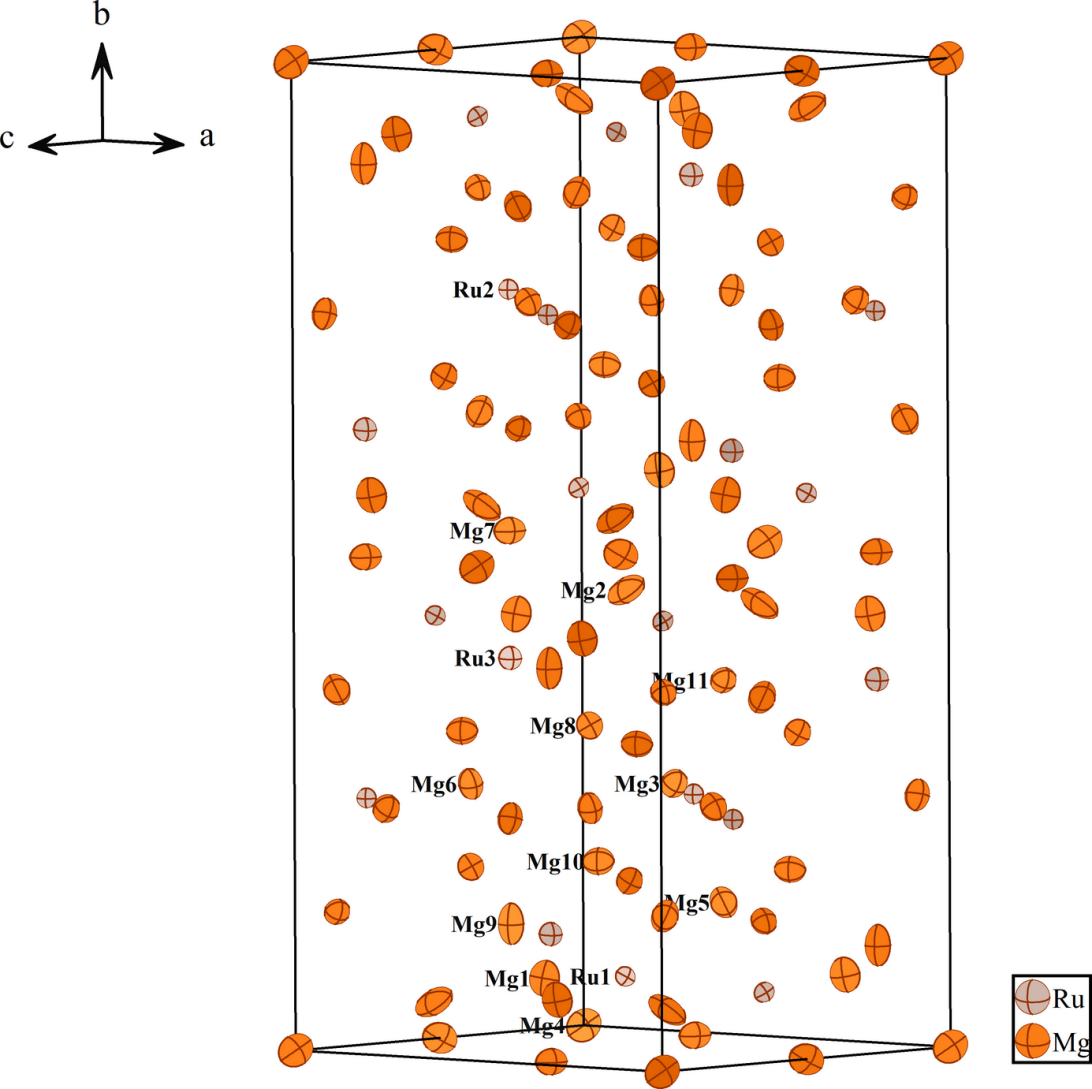
The unit cell of Mg_9_Ru_2_ with displacement ellipsoids at the 99% probability level. Labeled atoms correspond to those of the asymmetric unit

**Figure 2:**
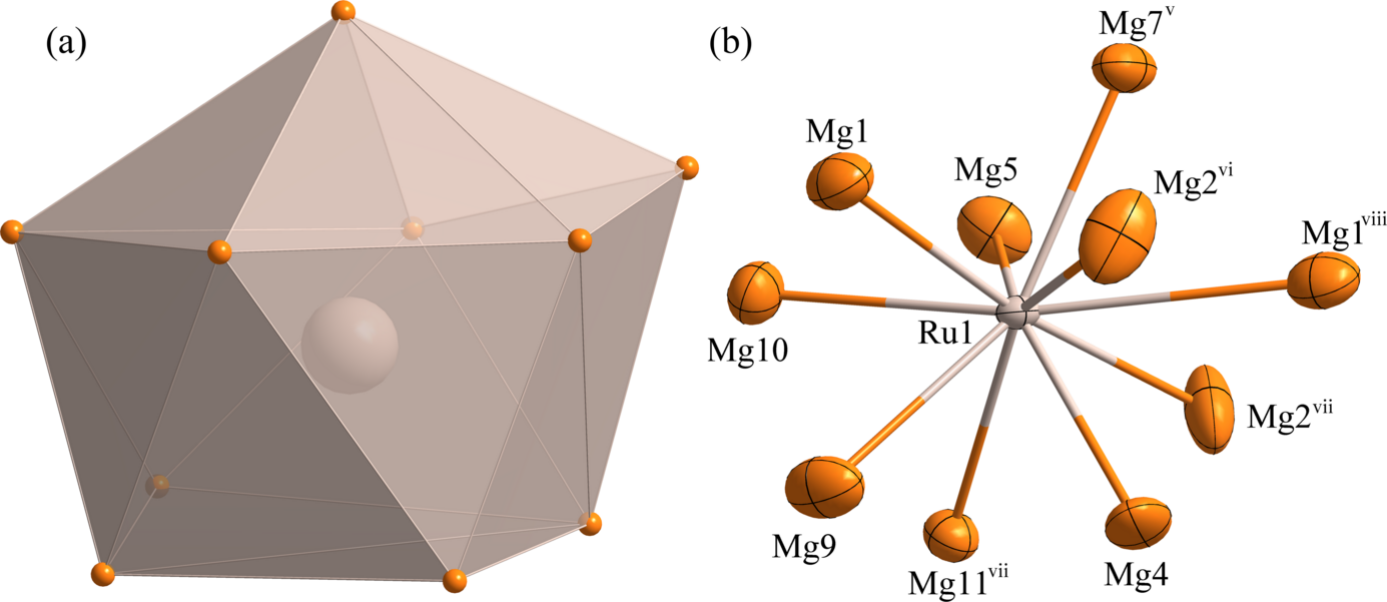
(*a*) The environment of the Ru1 atom at the 8 *f* site in polyhedral representation; (*b*) the environment of the Ru1 atom with displacement ellipsoids given at the 99% probability level. [Symmetry codes: (v) *x* + 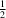, *y* − 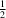, *z*; (vi) −*x* + 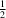, *y* − 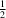, −*z* + 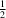; (vii) −*x* + 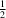, −*y* + 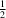, −*z*; (viii) *x*, −*y*, *z* − 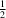.]

**Figure 5:**
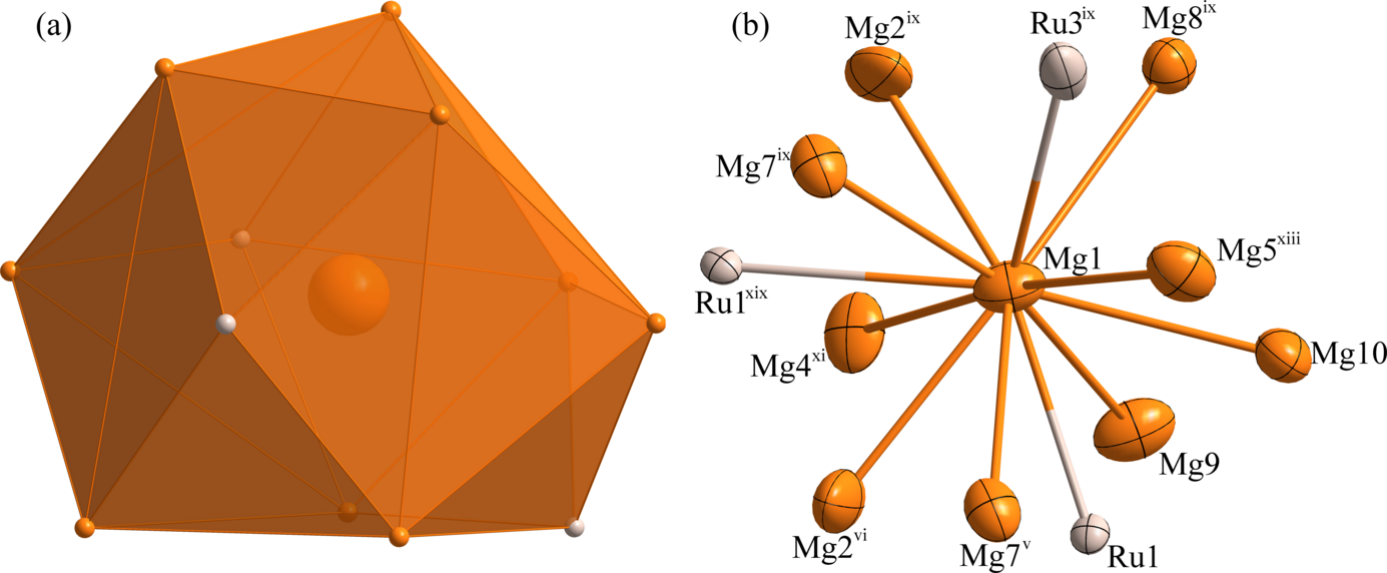
(*a*) The environment of the Mg1 atom at the 8 *f* site; (*b*) the environment of the Mg1 atom with displacement ellipsoids given at the 99% probability level. [Symmetry codes: (v) *x* + 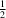, *y* − 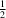, *z*; (vi) −*x* + 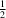, *y* − 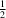, −*z* + 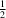; (ix) −*x* + 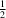, −*y* + 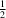, −*z* + 1; (xi) −*x*, *y*, −*z* + 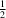; (xiii) −*x* + 1, *y*, −*z* + 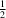; (xix) *x*, −*y*, *z* + 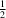.]

Fig. 1 ▸ illustrates the atomic distribution within the unit cell of Mg_9_Ru_2_. The environments of the Ru1, Ru2 and Ru3 sites are shown in Figs. 2 ▸, 3 ▸ and 4 ▸, respectively. The Ru1 atom is located at a general site (multiplicity 8, Wyckoff letter *f*) and is surrounded by ten Mg atoms, with the shortest Ru—Mg separation of 2.7269 (3) Å for Ru1—Mg4. The Ru2 and Ru3 atoms both occupy a site with symmetry 2 (4 *e*). They are surrounded by twelve and eleven Mg atoms, respectively. Here the shortest Ru—Mg separations are Ru2—Mg3 = 2.81020 (9) Å and Ru3—Mg1 = 2.8617 (15) Å. The environments of the Mg1 and Mg3 sites are shown in Figs. 5 ▸ and 6 ▸, respectively. The Mg1 atom occupies a general site and is surrounded by nine Mg atoms and three Ru atoms, with the shortest Mg—Ru separation of Mg1—Ru1 = 2.8552 (15) Å. The Mg3 atom is located at a site with symmetry 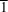 (4 *c*) and is pairwise surrounded by six atoms, Mg5, Mg6, Mg8, Mg10, Mg11, and Ru2, defining the center of an icosa­hedron.

## Synthesis and crystallization

High-purity elements magnesium (indicated purity 99.9%; 0.1785 g), ruthenium (indicated purity: 99.9%; 0.7421 g) and boron (indicated purity: 99.9%; 0.0793 g) with a stoichiometric ratio of 1:1:1 were mixed and ground in an agate mortar for 40 min. The resulting powder was then placed in a cemented carbide grinding mold with a diameter of 5 mm and pressed into a block at about 4 MPa for three minutes. Cylindrical blocks without deformation and cracks were obtained. Details of high pressure sinter­ing experiments using six-anvil high-temperature and high-pressure equipment can be found elsewhere (Liu & Fan, 2018 ▸). The sample was further pressurized to 6 GPa and heated to 1273 K for 40 min, and then quickly cooled to room temperature by turning off the furnace power. A selected single-crystal was mounted on a glass fiber for X-ray diffraction measurement.

## Refinement

Crystal data, data collection and structure refinement details are summarized in Table 1 ▸. The stoichiometric composition of the Mg_9_Ru_2_ phase aligns closely with the elemental ratios determined *via* energy-dispersive X-ray spectroscopy (EDX) analysis (see Table S1 of the supporting information). For better comparison with the Mg_9_Ru_2_ structure, the labelling scheme and atomic coordinates were adapted from isotypic In_1.9_Ir_3_Mg_17.1_ (Hlukhyy & Pöttgen, 2005 ▸). Ru1, Ru2 and Ru3 correspond to the positions of Ir1, Ir2 and In1, respectively, in the In_1.9_Ir_3_Mg_17.1_ phase, and all the positions of Mg atoms correspond to each other. For the Mg_9_Ru_2_ phase, the maximum and minimum residual electron densities in the final difference map are located 1.67 Å from site Ru1 and 0.66 Å from site Mg9, respectively.

## Supplementary Material

Crystal structure: contains datablock(s) I. DOI: 10.1107/S2414314625003050/wm4227sup1.cif

Structure factors: contains datablock(s) I. DOI: 10.1107/S2414314625003050/wm4227Isup2.hkl

Supporting information file. DOI: 10.1107/S2414314625003050/wm4227sup3.docx

CCDC reference: 2440944

Additional supporting information:  crystallographic information; 3D view; checkCIF report

## Figures and Tables

**Figure 3:**
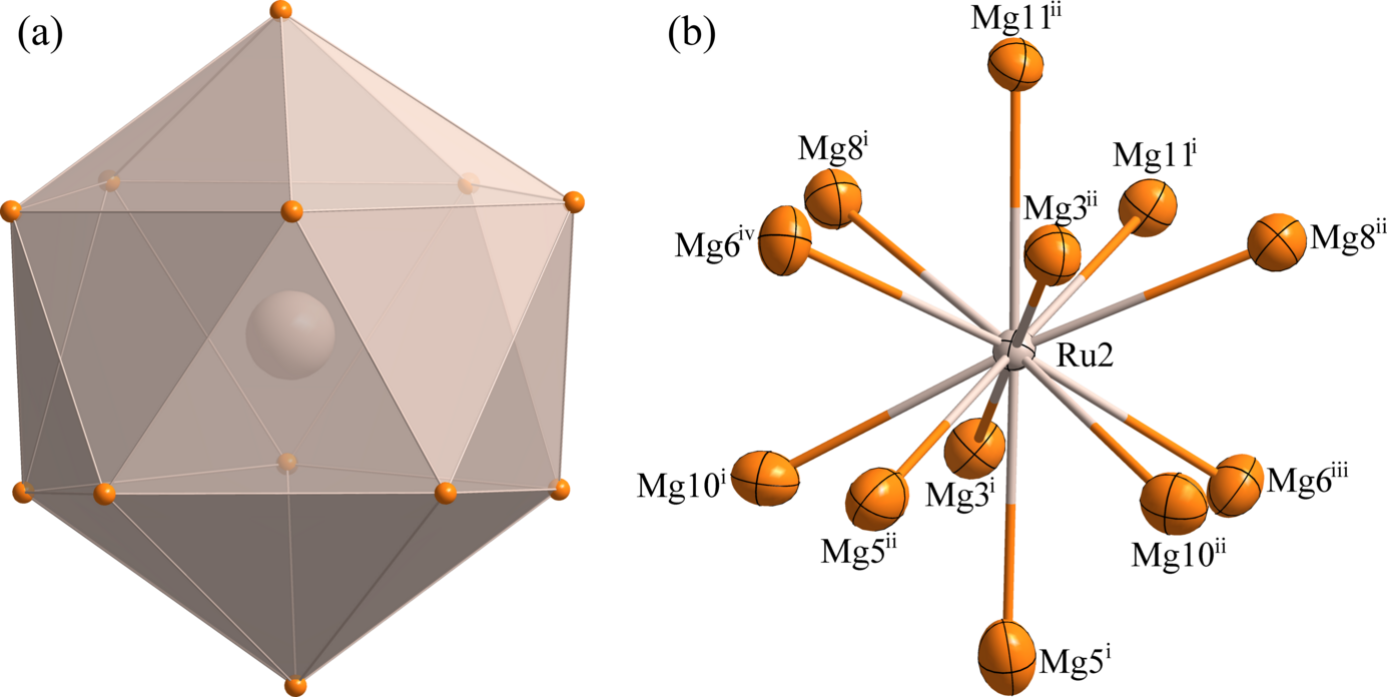
(*a*) The environment of the Ru2 atom at the 4 *e* site; (*b*) the environment of the Ru2 atom with displacement ellipsoids given at the 99% probability level. [Symmetry codes:(i) *x* − 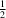, *y* + 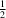, *z*; (ii) −*x* + 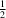, *y* + 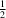, −*z* + 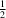; (iii) *x*, −*y* + 1, *z* − 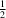; (iv) −*x*, −*y* + 1, −*z* + 1.]

**Figure 4:**
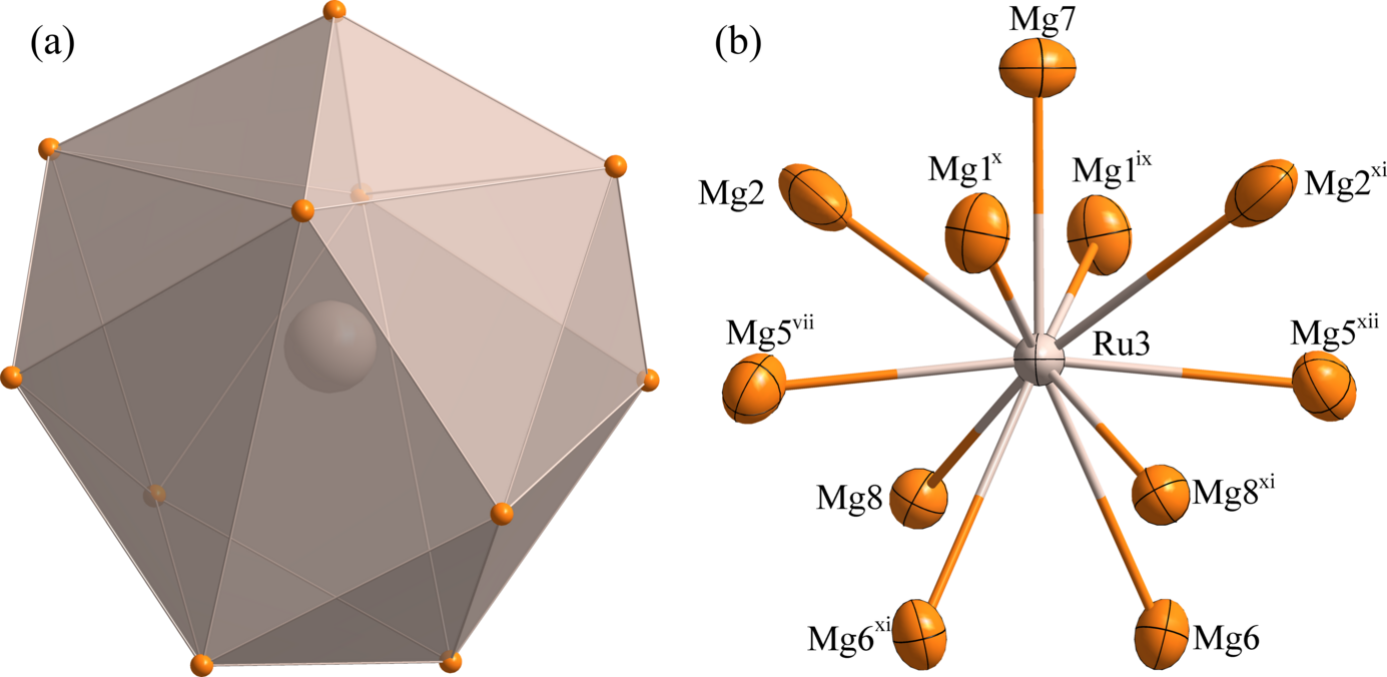
(*a*) The environment of the Ru3 atom at the 4 *e* site; (*b*) the environment of the Ru3 atom with displacement ellipsoids given at the 99% probability level. [Symmetry codes:(vii) −*x* + 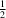, −*y* + 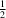, −*z*; (ix) −*x* + 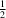, −*y* + 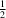, −*z* + 1; (*x*) *x* − 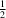, −*y* + 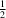, *z* − 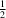; (xi) −*x*, *y*, −*z* + 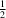; (xii) *x* − 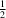, −*y* + 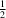, *z* + 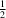.]

**Figure 6:**
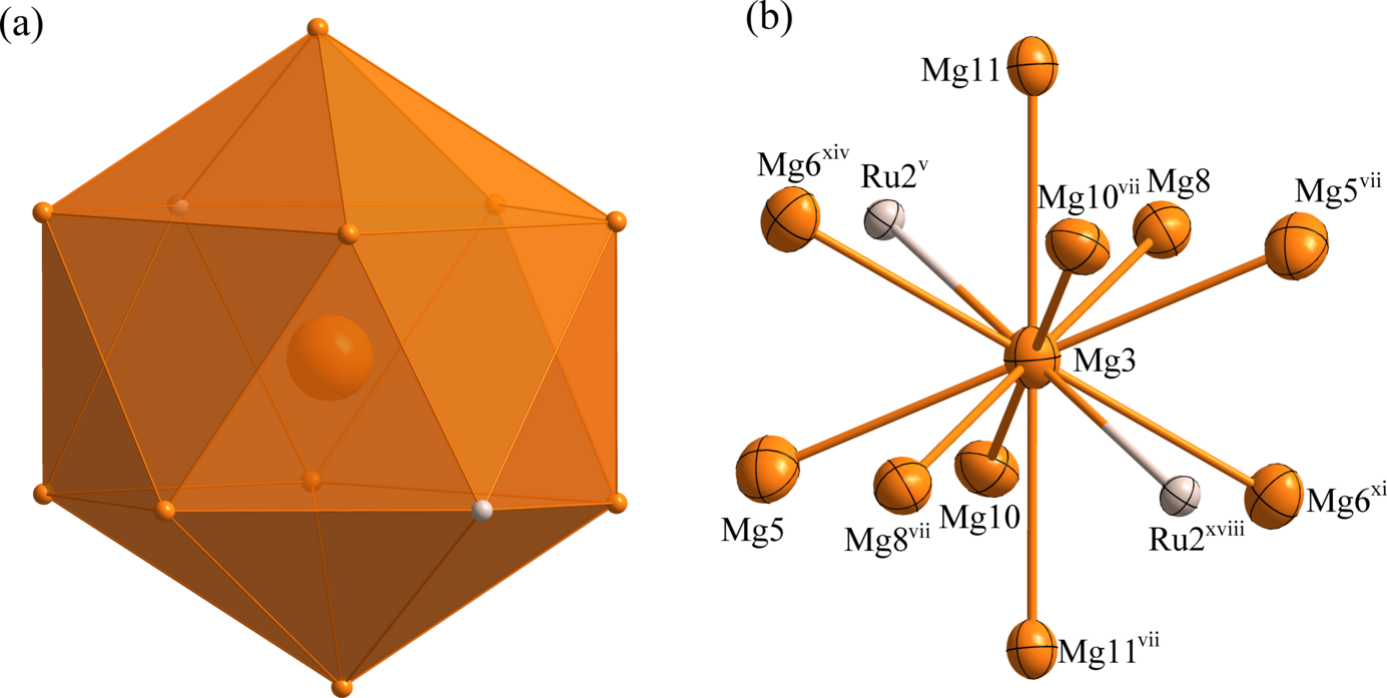
(*a*) The environment of the Mg3 atom at the 4 *c* site; (*b*) the environment of the Mg3 atom with displacement ellipsoids given at the 99% probability level. [Symmetry codes:(v) *x* + 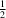, *y* − 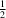, *z*; (vii) −*x* + 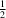, −*y* + 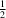, −*z*; (xi) −*x*, *y*, −*z* + 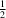; (xiv) *x* + 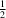, −*y* + 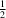, *z* − 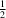; (xviii) −*x*, −*y* + 1, −*z*.]

**Table 1:** Experimental details

Crystal data	
Chemical formula	Mg_9_Ru_2_
*M*_r_	420.93
Crystal system, space group	Monoclinic, *C*2/*c*
Temperature (K)	296
*a*, *b*, *c* (Å)	9.8976 (4), 22.3449 (10), 8.6049 (3)
β (°)	105.549 (1)
*V* (Å^3^)	1833.42 (13)
*Z*	8
Radiation type	Mo *K*α
μ (mm^−1^)	3.83
Crystal size (mm)	0.14 × 0.10 × 0.02

Data collection	
Diffractometer	Bruker D8 Venture Photon 100 CMOS
Absorption correction	Multi-scan (*SADABS*; Krause *et al.*, 2015 ▸)
*T*_min_, *T*_max_	0.639, 0.746
No. of measured, independent and observed [*I* > 2σ(*I*)] reflections	29030, 2125, 1685
*R*_int_	0.095
(sin θ/λ)_max_ (Å^−1^)	0.650

Refinement	
*R*[*F*^2^ > 2σ(*F*^2^)], *wR*(*F*^2^), *S*	0.031, 0.059, 1.10
No. of reflections	2125
No. of parameters	105
Δρ_max_, Δρ_min_ (e Å^−3^)	1.06, −0.90

Computer programs: *APEX3* and *SAINT* (Bruker, 2015 ▸), *SHELXT* (Sheldrick, 2015*a* ▸), *SHELXL* (Sheldrick, 2015*b* ▸), *DIAMOND* (Brandenburg & Putz, 2017 ▸) and *publCIF* (Westrip, 2010 ▸).

## full crystallographic data

### Nonamagnesium diruthenium Crystal data

**Table:**

Mg_9_Ru_2_	*F*(000) = 1568
*M**_r_* = 420.93	*D*_x_ = 3.050 Mg m^−^^3^
Monoclinic, *C*2/*c*	Mo *K*α radiation, λ = 0.71073 Å
*a* = 9.8976 (4) Å	Cell parameters from 7618 reflections
*b* = 22.3449 (10) Å	θ = 2.3–27.5°
*c* = 8.6049 (3) Å	µ = 3.83 mm^−^^1^
β = 105.549 (1)°	*T* = 296 K
*V* = 1833.42 (13) Å^3^	Lump, gray
*Z* = 8	0.14 × 0.10 × 0.02 mm

### Nonamagnesium diruthenium Data collection

**Table:**

Bruker D8 Venture Photon 100 CMOS diffractometer	1685 reflections with *I* > 2σ(*I*)
phi and ω scans	*R*_int_ = 0.095
Absorption correction: multi-scan (SADABS; Krause *et al.*, 2015)	θ_max_ = 27.5°, θ_min_ = 2.3°
*T*_min_ = 0.639, *T*_max_ = 0.746	*h* = −12→12
29030 measured reflections	*k* = −28→28
2125 independent reflections	*l* = −11→11

### Nonamagnesium diruthenium Refinement

**Table:**

Refinement on *F*^2^	105 parameters
Least-squares matrix: full	0 restraints
*R*[*F*^2^ > 2σ(*F*^2^)] = 0.031	*w* = 1/[σ^2^(*F*_o_^2^) + (0.0178*P*)^2^ + 7.9309*P*] where *P* = (*F*_o_^2^ + 2*F*_c_^2^)/3
*wR*(*F*^2^) = 0.059	(Δ/σ)_max_ = 0.001
*S* = 1.10	Δρ_max_ = 1.06 e Å^−^^3^
2125 reflections	Δρ_min_ = −0.90 e Å^−^^3^

### Nonamagnesium diruthenium Special details

**Table:**

Geometry. All esds (except the esd in the dihedral angle between two l.s. planes) are estimated using the full covariance matrix. The cell esds are taken into account individually in the estimation of esds in distances, angles and torsion angles; correlations between esds in cell parameters are only used when they are defined by crystal symmetry. An approximate (isotropic) treatment of cell esds is used for estimating esds involving l.s. planes.

### Nonamagnesium diruthenium Fractional atomic coordinates and isotropic or equivalent isotropic displacement parameters (Å^2^)

**Table:**

	*x*	*y*	*z*	*U*_iso_*/*U*_eq_	
Ru1	0.23887 (3)	0.05857 (2)	0.15867 (4)	0.00565 (9)	
Ru2	0.000000	0.75111 (2)	0.250000	0.00573 (12)	
Ru3	0.000000	0.37787 (2)	0.250000	0.01233 (13)	
Mg1	0.28646 (16)	0.06659 (7)	0.50027 (17)	0.0149 (3)	
Mg2	0.26554 (18)	0.45064 (7)	0.18284 (19)	0.0200 (4)	
Mg3	0.250000	0.250000	0.000000	0.0120 (4)	
Mg4	0.000000	0.000000	0.000000	0.0163 (5)	
Mg5	0.41829 (16)	0.13455 (7)	0.04409 (19)	0.0153 (3)	
Mg6	0.03146 (16)	0.25579 (7)	0.42870 (17)	0.0127 (3)	
Mg7	0.000000	0.50705 (9)	0.250000	0.0128 (5)	
Mg8	0.26330 (16)	0.31691 (7)	0.30981 (18)	0.0119 (3)	
Mg9	0.000000	0.10898 (11)	0.250000	0.0175 (5)	
Mg10	0.26689 (16)	0.17899 (7)	0.28609 (18)	0.0132 (3)	
Mg11	0.43520 (15)	0.36005 (6)	0.06331 (17)	0.0103 (3)	

### Nonamagnesium diruthenium Atomic displacement parameters (Å^2^)

**Table:**

	*U*^11^	*U*^22^	*U*^33^	*U*^12^	*U*^13^	*U*^23^
Ru1	0.00419 (17)	0.00620 (17)	0.00585 (17)	−0.00009 (14)	0.00009 (13)	0.00059 (14)
Ru2	0.0055 (2)	0.0063 (2)	0.0051 (2)	0.000	0.00101 (18)	0.000
Ru3	0.0173 (3)	0.0086 (3)	0.0142 (3)	0.000	0.0096 (2)	0.000
Mg1	0.0127 (8)	0.0202 (8)	0.0107 (8)	0.0001 (7)	0.0013 (6)	−0.0014 (7)
Mg2	0.0321 (10)	0.0147 (9)	0.0139 (8)	0.0077 (7)	0.0076 (8)	−0.0025 (7)
Mg3	0.0123 (11)	0.0127 (11)	0.0110 (10)	0.0011 (9)	0.0034 (9)	−0.0001 (9)
Mg4	0.0077 (11)	0.0167 (11)	0.0219 (13)	−0.0028 (9)	−0.0003 (10)	−0.0058 (10)
Mg5	0.0144 (8)	0.0159 (8)	0.0173 (8)	0.0005 (7)	0.0070 (7)	0.0036 (6)
Mg6	0.0125 (8)	0.0157 (8)	0.0106 (8)	−0.0001 (7)	0.0045 (6)	0.0016 (6)
Mg7	0.0087 (11)	0.0112 (11)	0.0165 (12)	0.000	0.0001 (9)	0.000
Mg8	0.0125 (8)	0.0116 (7)	0.0123 (8)	−0.0001 (6)	0.0044 (7)	0.0005 (6)
Mg9	0.0116 (12)	0.0278 (13)	0.0140 (12)	0.000	0.0050 (10)	0.000
Mg10	0.0145 (8)	0.0112 (8)	0.0132 (8)	−0.0012 (6)	0.0025 (7)	−0.0019 (6)
Mg11	0.0102 (8)	0.0106 (7)	0.0099 (8)	0.0018 (6)	0.0024 (6)	0.0010 (6)

### Nonamagnesium diruthenium Geometric parameters (Å, º)

**Table:**

Ru1—Mg4	2.7269 (3)	Mg2—Mg11	2.983 (2)
Ru1—Mg7^i^	2.7448 (9)	Mg2—Mg7	3.1056 (18)
Ru1—Mg2^ii^	2.7768 (16)	Mg2—Mg8	3.184 (2)
Ru1—Mg5	2.8190 (15)	Mg2—Mg4^vi^	3.2641 (17)
Ru1—Mg1	2.8552 (15)	Mg2—Mg4^xiv^	3.3218 (17)
Ru1—Mg11^iii^	2.8565 (14)	Mg3—Mg10^iii^	2.8957 (15)
Ru1—Mg10	2.8908 (15)	Mg3—Mg10	2.8957 (15)
Ru1—Mg9	2.9107 (10)	Mg3—Mg6^xv^	3.0116 (15)
Ru1—Mg2^iii^	2.9345 (16)	Mg3—Mg6^xi^	3.0116 (15)
Ru1—Mg1^iv^	3.2010 (16)	Mg3—Mg11^iii^	3.0279 (15)
Ru2—Mg3^v^	2.8102 (1)	Mg3—Mg11	3.0279 (15)
Ru2—Mg3^vi^	2.8102 (1)	Mg3—Mg8^iii^	3.0283 (15)
Ru2—Mg6^vii^	2.8681 (14)	Mg3—Mg8	3.0284 (15)
Ru2—Mg6^viii^	2.8681 (14)	Mg3—Mg5	3.0388 (15)
Ru2—Mg11^vi^	2.8923 (15)	Mg3—Mg5^iii^	3.0388 (15)
Ru2—Mg11^v^	2.8923 (15)	Mg4—Mg9^xvi^	3.2492 (18)
Ru2—Mg10^v^	2.8984 (16)	Mg4—Mg9	3.2493 (18)
Ru2—Mg10^vi^	2.8984 (16)	Mg4—Mg11^iii^	3.2673 (14)
Ru2—Mg8^vi^	2.9260 (15)	Mg4—Mg11^xvii^	3.2673 (14)
Ru2—Mg8^v^	2.9260 (15)	Mg5—Mg6^xv^	2.973 (2)
Ru2—Mg5^v^	3.1318 (16)	Mg5—Mg10	3.040 (2)
Ru2—Mg5^vi^	3.1318 (16)	Mg5—Mg10^xiii^	3.223 (2)
Ru3—Mg1^ix^	2.8617 (15)	Mg5—Mg8^iii^	3.278 (2)
Ru3—Mg1^x^	2.8617 (15)	Mg5—Mg7^i^	3.338 (2)
Ru3—Mg8	2.8624 (15)	Mg5—Mg11^iii^	3.373 (2)
Ru3—Mg8^xi^	2.8624 (15)	Mg6—Mg6^xi^	2.970 (3)
Ru3—Mg5^iii^	2.8692 (15)	Mg6—Mg8^ix^	3.062 (2)
Ru3—Mg5^xii^	2.8692 (15)	Mg6—Mg8	3.072 (2)
Ru3—Mg7	2.886 (2)	Mg6—Mg10^ix^	3.083 (2)
Ru3—Mg6^xi^	3.1059 (15)	Mg6—Mg11^xii^	3.089 (2)
Ru3—Mg6	3.1059 (15)	Mg6—Mg8^xi^	3.373 (2)
Ru3—Mg2^xi^	3.2710 (18)	Mg6—Mg10	3.378 (2)
Ru3—Mg2	3.2710 (18)	Mg6—Mg10^xi^	3.486 (2)
Mg1—Mg2^ix^	2.933 (2)	Mg8—Mg11^xiii^	3.046 (2)
Mg1—Mg2^ii^	3.004 (2)	Mg8—Mg10	3.089 (2)
Mg1—Mg7^ix^	3.0590 (18)	Mg8—Mg11	3.203 (2)
Mg1—Mg10	3.090 (2)	Mg9—Mg10^xi^	3.014 (2)
Mg1—Mg8^ix^	3.181 (2)	Mg9—Mg10	3.014 (2)
Mg1—Mg4^xi^	3.2015 (15)	Mg9—Mg11^xii^	3.0143 (15)
Mg1—Mg9	3.2094 (16)	Mg9—Mg11^iii^	3.0143 (15)
Mg1—Mg5^xiii^	3.404 (2)	Mg10—Mg11^iii^	3.252 (2)
Mg1—Mg7^i^	3.6491 (17)	Mg11—Mg11^xiii^	3.124 (3)
Mg2—Mg5^iii^	2.974 (2)		
			
Mg4—Ru1—Mg7^i^	124.44 (4)	Ru3^iii^—Mg5—Ru2^i^	119.23 (5)
Mg4—Ru1—Mg2^ii^	72.75 (4)	Mg6^xv^—Mg5—Ru2^i^	55.97 (4)
Mg7^i^—Ru1—Mg2^ii^	68.45 (5)	Mg2^iii^—Mg5—Ru2^i^	157.46 (6)
Mg4—Ru1—Mg5	131.06 (3)	Mg3—Mg5—Ru2^i^	54.16 (3)
Mg7^i^—Ru1—Mg5	73.72 (5)	Mg10—Mg5—Ru2^i^	56.00 (4)
Mg2^ii^—Ru1—Mg5	142.11 (5)	Ru1—Mg5—Mg10^xiii^	130.54 (6)
Mg4—Ru1—Mg1	115.29 (3)	Ru3^iii^—Mg5—Mg10^xiii^	87.87 (5)
Mg7^i^—Ru1—Mg1	81.30 (3)	Mg6^xv^—Mg5—Mg10^xiii^	59.51 (5)
Mg2^ii^—Ru1—Mg1	64.46 (5)	Mg2^iii^—Mg5—Mg10^xiii^	147.34 (7)
Mg5—Ru1—Mg1	112.13 (5)	Mg3—Mg5—Mg10^xiii^	103.06 (5)
Mg4—Ru1—Mg11^iii^	71.59 (3)	Mg10—Mg5—Mg10^xiii^	100.54 (6)
Mg7^i^—Ru1—Mg11^iii^	144.23 (4)	Ru2^i^—Mg5—Mg10^xiii^	54.24 (4)
Mg2^ii^—Ru1—Mg11^iii^	142.68 (5)	Ru1—Mg5—Mg8^iii^	106.52 (5)
Mg5—Ru1—Mg11^iii^	72.92 (4)	Ru3^iii^—Mg5—Mg8^iii^	55.02 (4)
Mg1—Ru1—Mg11^iii^	123.50 (4)	Mg6^xv^—Mg5—Mg8^iii^	65.10 (5)
Mg4—Ru1—Mg10	128.23 (3)	Mg2^iii^—Mg5—Mg8^iii^	61.00 (5)
Mg7^i^—Ru1—Mg10	107.13 (5)	Mg3—Mg5—Mg8^iii^	57.14 (4)
Mg2^ii^—Ru1—Mg10	129.40 (5)	Mg10—Mg5—Mg8^iii^	106.26 (6)
Mg5—Ru1—Mg10	64.32 (5)	Ru2^i^—Mg5—Mg8^iii^	104.15 (5)
Mg1—Ru1—Mg10	65.07 (4)	Mg10^xiii^—Mg5—Mg8^iii^	122.81 (6)
Mg11^iii^—Ru1—Mg10	68.92 (4)	Ru1—Mg5—Mg7^i^	52.12 (3)
Mg4—Ru1—Mg9	70.30 (3)	Ru3^iii^—Mg5—Mg7^i^	107.46 (5)
Mg7^i^—Ru1—Mg9	148.832 (15)	Mg6^xv^—Mg5—Mg7^i^	145.21 (6)
Mg2^ii^—Ru1—Mg9	94.74 (5)	Mg2^iii^—Mg5—Mg7^i^	79.67 (5)
Mg5—Ru1—Mg9	119.66 (5)	Mg3—Mg5—Mg7^i^	146.88 (5)
Mg1—Ru1—Mg9	67.64 (3)	Mg10—Mg5—Mg7^i^	90.52 (5)
Mg11^iii^—Ru1—Mg9	63.01 (4)	Ru2^i^—Mg5—Mg7^i^	114.86 (5)
Mg10—Ru1—Mg9	62.59 (5)	Mg10^xiii^—Mg5—Mg7^i^	87.43 (5)
Mg4—Ru1—Mg2^iii^	71.75 (4)	Mg8^iii^—Mg5—Mg7^i^	140.36 (6)
Mg7^i^—Ru1—Mg2^iii^	91.00 (3)	Ru1—Mg5—Mg11^iii^	54.05 (4)
Mg2^ii^—Ru1—Mg2^iii^	115.66 (3)	Ru3^iii^—Mg5—Mg11^iii^	106.18 (5)
Mg5—Ru1—Mg2^iii^	62.20 (5)	Mg6^xv^—Mg5—Mg11^iii^	109.57 (6)
Mg1—Ru1—Mg2^iii^	171.67 (5)	Mg2^iii^—Mg5—Mg11^iii^	55.65 (5)
Mg11^iii^—Ru1—Mg2^iii^	62.00 (4)	Mg3—Mg5—Mg11^iii^	56.07 (4)
Mg10—Ru1—Mg2^iii^	114.79 (4)	Mg10—Mg5—Mg11^iii^	60.69 (5)
Mg9—Ru1—Mg2^iii^	120.16 (4)	Ru2^i^—Mg5—Mg11^iii^	102.53 (5)
Mg4—Ru1—Mg1^iv^	64.80 (3)	Mg10^xiii^—Mg5—Mg11^iii^	156.77 (6)
Mg7^i^—Ru1—Mg1^iv^	61.36 (4)	Mg8^iii^—Mg5—Mg11^iii^	57.55 (4)
Mg2^ii^—Ru1—Mg1^iv^	58.24 (4)	Mg7^i^—Mg5—Mg11^iii^	105.18 (5)
Mg5—Ru1—Mg1^iv^	101.47 (4)	Ru1—Mg5—Mg1^xiii^	114.25 (6)
Mg1—Ru1—Mg1^iv^	119.39 (4)	Ru3^iii^—Mg5—Mg1^xiii^	53.45 (4)
Mg11^iii^—Ru1—Mg1^iv^	113.75 (4)	Mg6^xv^—Mg5—Mg1^xiii^	85.33 (5)
Mg10—Ru1—Mg1^iv^	164.73 (4)	Mg2^iii^—Mg5—Mg1^xiii^	91.91 (6)
Mg9—Ru1—Mg1^iv^	132.53 (5)	Mg3—Mg5—Mg1^xiii^	145.38 (6)
Mg2^iii^—Ru1—Mg1^iv^	58.45 (4)	Mg10—Mg5—Mg1^xiii^	144.90 (6)
Mg3^v^—Ru2—Mg3^vi^	178.993 (19)	Ru2^i^—Mg5—Mg1^xiii^	109.58 (5)
Mg3^v^—Ru2—Mg6^vii^	64.05 (3)	Mg10^xiii^—Mg5—Mg1^xiii^	55.51 (4)
Mg3^vi^—Ru2—Mg6^vii^	115.89 (3)	Mg8^iii^—Mg5—Mg1^xiii^	108.47 (6)
Mg3^v^—Ru2—Mg6^viii^	115.89 (3)	Mg7^i^—Mg5—Mg1^xiii^	65.52 (4)
Mg3^vi^—Ru2—Mg6^viii^	64.05 (3)	Mg11^iii^—Mg5—Mg1^xiii^	147.54 (6)
Mg6^vii^—Ru2—Mg6^viii^	173.84 (6)	Ru2^viii^—Mg6—Mg6^xi^	162.08 (8)
Mg3^v^—Ru2—Mg11^vi^	116.82 (3)	Ru2^viii^—Mg6—Mg5^xii^	64.81 (4)
Mg3^vi^—Ru2—Mg11^vi^	64.12 (3)	Mg6^xi^—Mg6—Mg5^xii^	110.65 (5)
Mg6^vii^—Ru2—Mg11^vi^	121.02 (4)	Ru2^viii^—Mg6—Mg3^xi^	57.04 (3)
Mg6^viii^—Ru2—Mg11^vi^	64.85 (4)	Mg6^xi^—Mg6—Mg3^xi^	105.21 (7)
Mg3^v^—Ru2—Mg11^v^	64.12 (3)	Mg5^xii^—Mg6—Mg3^xi^	61.02 (4)
Mg3^vi^—Ru2—Mg11^v^	116.82 (3)	Ru2^viii^—Mg6—Mg8^ix^	59.02 (4)
Mg6^vii^—Ru2—Mg11^v^	64.85 (4)	Mg6^xi^—Mg6—Mg8^ix^	132.42 (6)
Mg6^viii^—Ru2—Mg11^v^	121.02 (4)	Mg5^xii^—Mg6—Mg8^ix^	115.27 (6)
Mg11^vi^—Ru2—Mg11^v^	65.37 (6)	Mg3^xi^—Mg6—Mg8^ix^	107.17 (5)
Mg3^v^—Ru2—Mg10^v^	60.94 (3)	Ru2^viii^—Mg6—Mg8	128.96 (6)
Mg3^vi^—Ru2—Mg10^v^	118.42 (3)	Mg6^xi^—Mg6—Mg8	67.86 (6)
Mg6^vii^—Ru2—Mg10^v^	111.63 (4)	Mg5^xii^—Mg6—Mg8	97.28 (6)
Mg6^viii^—Ru2—Mg10^v^	64.63 (4)	Mg3^xi^—Mg6—Mg8	154.33 (6)
Mg11^vi^—Ru2—Mg10^v^	118.21 (4)	Mg8^ix^—Mg6—Mg8	94.18 (5)
Mg11^v^—Ru2—Mg10^v^	117.58 (4)	Ru2^viii^—Mg6—Mg10^ix^	58.16 (4)
Mg3^v^—Ru2—Mg10^vi^	118.42 (3)	Mg6^xi^—Mg6—Mg10^ix^	137.36 (7)
Mg3^vi^—Ru2—Mg10^vi^	60.94 (3)	Mg5^xii^—Mg6—Mg10^ix^	64.28 (5)
Mg6^vii^—Ru2—Mg10^vi^	64.63 (4)	Mg3^xi^—Mg6—Mg10^ix^	107.15 (5)
Mg6^viii^—Ru2—Mg10^vi^	111.63 (4)	Mg8^ix^—Mg6—Mg10^ix^	60.36 (5)
Mg11^vi^—Ru2—Mg10^vi^	117.58 (4)	Mg8—Mg6—Mg10^ix^	70.91 (5)
Mg11^v^—Ru2—Mg10^vi^	118.21 (4)	Ru2^viii^—Mg6—Mg11^xii^	57.95 (4)
Mg10^v^—Ru2—Mg10^vi^	112.45 (6)	Mg6^xi^—Mg6—Mg11^xii^	112.85 (5)
Mg3^v^—Ru2—Mg8^vi^	116.87 (3)	Mg5^xii^—Mg6—Mg11^xii^	112.56 (6)
Mg3^vi^—Ru2—Mg8^vi^	63.69 (3)	Mg3^xi^—Mg6—Mg11^xii^	59.50 (4)
Mg6^vii^—Ru2—Mg8^vi^	63.80 (4)	Mg8^ix^—Mg6—Mg11^xii^	59.37 (5)
Mg6^viii^—Ru2—Mg8^vi^	119.70 (4)	Mg8—Mg6—Mg11^xii^	146.15 (7)
Mg11^vi^—Ru2—Mg8^vi^	66.80 (4)	Mg10^ix^—Mg6—Mg11^xii^	107.25 (6)
Mg11^v^—Ru2—Mg8^vi^	63.14 (4)	Ru2^viii^—Mg6—Ru3	120.14 (5)
Mg10^v^—Ru2—Mg8^vi^	174.95 (4)	Mg6^xi^—Mg6—Ru3	61.44 (3)
Mg10^vi^—Ru2—Mg8^vi^	64.06 (4)	Mg5^xii^—Mg6—Ru3	56.27 (4)
Mg3^v^—Ru2—Mg8^v^	63.69 (3)	Mg3^xi^—Mg6—Ru3	99.39 (4)
Mg3^vi^—Ru2—Mg8^v^	116.87 (3)	Mg8^ix^—Mg6—Ru3	142.04 (6)
Mg6^vii^—Ru2—Mg8^v^	119.70 (4)	Mg8—Mg6—Ru3	55.20 (4)
Mg6^viii^—Ru2—Mg8^v^	63.80 (4)	Mg10^ix^—Mg6—Ru3	86.36 (5)
Mg11^vi^—Ru2—Mg8^v^	63.14 (4)	Mg11^xii^—Mg6—Ru3	157.16 (6)
Mg11^v^—Ru2—Mg8^v^	66.80 (4)	Ru2^viii^—Mg6—Mg8^xi^	107.97 (5)
Mg10^v^—Ru2—Mg8^v^	64.06 (4)	Mg6^xi^—Mg6—Mg8^xi^	57.51 (5)
Mg10^vi^—Ru2—Mg8^v^	174.95 (4)	Mg5^xii^—Mg6—Mg8^xi^	61.82 (5)
Mg8^vi^—Ru2—Mg8^v^	119.67 (6)	Mg3^xi^—Mg6—Mg8^xi^	56.29 (4)
Mg3^v^—Ru2—Mg5^v^	61.23 (3)	Mg8^ix^—Mg6—Mg8^xi^	163.14 (7)
Mg3^vi^—Ru2—Mg5^v^	117.82 (3)	Mg8—Mg6—Mg8^xi^	102.64 (6)
Mg6^vii^—Ru2—Mg5^v^	59.22 (4)	Mg10^ix^—Mg6—Mg8^xi^	124.25 (6)
Mg6^viii^—Ru2—Mg5^v^	114.99 (4)	Mg11^xii^—Mg6—Mg8^xi^	105.31 (6)
Mg11^vi^—Ru2—Mg5^v^	177.90 (4)	Ru3—Mg6—Mg8^xi^	52.24 (3)
Mg11^v^—Ru2—Mg5^v^	113.61 (4)	Ru2^viii^—Mg6—Mg10	125.91 (5)
Mg10^v^—Ru2—Mg5^v^	60.40 (4)	Mg6^xi^—Mg6—Mg10	66.26 (5)
Mg10^vi^—Ru2—Mg5^v^	64.49 (4)	Mg5^xii^—Mg6—Mg10	153.92 (7)
Mg8^vi^—Ru2—Mg5^v^	114.57 (4)	Mg3^xi^—Mg6—Mg10	144.88 (6)
Mg8^v^—Ru2—Mg5^v^	114.83 (4)	Mg8^ix^—Mg6—Mg10	67.12 (5)
Mg3^v^—Ru2—Mg5^vi^	117.82 (3)	Mg8—Mg6—Mg10	56.99 (4)
Mg3^vi^—Ru2—Mg5^vi^	61.23 (3)	Mg10^ix^—Mg6—Mg10	99.61 (5)
Mg6^vii^—Ru2—Mg5^vi^	114.99 (4)	Mg11^xii^—Mg6—Mg10	91.30 (5)
Mg6^viii^—Ru2—Mg5^vi^	59.22 (4)	Ru3—Mg6—Mg10	104.69 (5)
Mg11^vi^—Ru2—Mg5^vi^	113.61 (4)	Mg8^xi^—Mg6—Mg10	123.57 (6)
Mg11^v^—Ru2—Mg5^vi^	177.90 (4)	Ru2^viii^—Mg6—Mg10^xi^	100.91 (5)
Mg10^v^—Ru2—Mg5^vi^	64.49 (4)	Mg6^xi^—Mg6—Mg10^xi^	62.50 (5)
Mg10^vi^—Ru2—Mg5^vi^	60.40 (4)	Mg5^xii^—Mg6—Mg10^xi^	103.96 (6)
Mg8^vi^—Ru2—Mg5^vi^	114.83 (4)	Mg3^xi^—Mg6—Mg10^xi^	52.31 (4)
Mg8^v^—Ru2—Mg5^vi^	114.57 (4)	Mg8^ix^—Mg6—Mg10^xi^	115.42 (6)
Mg5^v^—Ru2—Mg5^vi^	67.47 (6)	Mg8—Mg6—Mg10^xi^	130.12 (6)
Mg1^ix^—Ru3—Mg1^x^	128.61 (6)	Mg10^ix^—Mg6—Mg10^xi^	158.48 (7)
Mg1^ix^—Ru3—Mg8	67.53 (4)	Mg11^xii^—Mg6—Mg10^xi^	58.92 (4)
Mg1^x^—Ru3—Mg8	142.65 (4)	Ru3—Mg6—Mg10^xi^	102.22 (5)
Mg1^ix^—Ru3—Mg8^xi^	142.65 (4)	Mg8^xi^—Mg6—Mg10^xi^	53.50 (4)
Mg1^x^—Ru3—Mg8^xi^	67.53 (4)	Mg10—Mg6—Mg10^xi^	97.24 (6)
Mg8—Ru3—Mg8^xi^	123.16 (6)	Ru1^vi^—Mg7—Ru1^v^	130.40 (8)
Mg1^ix^—Ru3—Mg5^iii^	112.22 (4)	Ru1^vi^—Mg7—Ru3	114.80 (4)
Mg1^x^—Ru3—Mg5^iii^	72.89 (4)	Ru1^v^—Mg7—Ru3	114.80 (4)
Mg8—Ru3—Mg5^iii^	69.77 (4)	Ru1^vi^—Mg7—Mg1^x^	147.89 (3)
Mg8^xi^—Ru3—Mg5^iii^	104.69 (4)	Ru1^v^—Mg7—Mg1^x^	66.69 (3)
Mg1^ix^—Ru3—Mg5^xii^	72.89 (4)	Ru3—Mg7—Mg1^x^	57.46 (4)
Mg1^x^—Ru3—Mg5^xii^	112.22 (4)	Ru1^vi^—Mg7—Mg1^ix^	66.69 (3)
Mg8—Ru3—Mg5^xii^	104.69 (4)	Ru1^v^—Mg7—Mg1^ix^	147.89 (3)
Mg8^xi^—Ru3—Mg5^xii^	69.77 (4)	Ru3—Mg7—Mg1^ix^	57.46 (4)
Mg5^iii^—Ru3—Mg5^xii^	168.89 (6)	Mg1^x^—Mg7—Mg1^ix^	114.91 (8)
Mg1^ix^—Ru3—Mg7	64.30 (3)	Ru1^vi^—Mg7—Mg2	56.26 (3)
Mg1^x^—Ru3—Mg7	64.30 (3)	Ru1^v^—Mg7—Mg2	153.62 (3)
Mg8—Ru3—Mg7	118.42 (3)	Ru3—Mg7—Mg2	66.05 (5)
Mg8^xi^—Ru3—Mg7	118.42 (3)	Mg1^x^—Mg7—Mg2	96.36 (6)
Mg5^iii^—Ru3—Mg7	95.55 (3)	Mg1^ix^—Mg7—Mg2	56.81 (4)
Mg5^xii^—Ru3—Mg7	95.55 (3)	Ru1^vi^—Mg7—Mg2^xi^	153.62 (3)
Mg1^ix^—Ru3—Mg6^xi^	135.11 (4)	Ru1^v^—Mg7—Mg2^xi^	56.26 (3)
Mg1^x^—Ru3—Mg6^xi^	93.05 (4)	Ru3—Mg7—Mg2^xi^	66.05 (5)
Mg8—Ru3—Mg6^xi^	68.69 (4)	Mg1^x^—Mg7—Mg2^xi^	56.81 (4)
Mg8^xi^—Ru3—Mg6^xi^	61.79 (4)	Mg1^ix^—Mg7—Mg2^xi^	96.36 (6)
Mg5^iii^—Ru3—Mg6^xi^	59.53 (4)	Mg2—Mg7—Mg2^xi^	132.11 (9)
Mg5^xii^—Ru3—Mg6^xi^	109.70 (4)	Ru1^vi^—Mg7—Mg5^vi^	54.16 (4)
Mg7—Ru3—Mg6^xi^	151.44 (3)	Ru1^v^—Mg7—Mg5^vi^	82.50 (5)
Mg1^ix^—Ru3—Mg6	93.05 (4)	Ru3—Mg7—Mg5^vi^	148.60 (3)
Mg1^x^—Ru3—Mg6	135.11 (4)	Mg1^x^—Mg7—Mg5^vi^	148.81 (6)
Mg8—Ru3—Mg6	61.79 (4)	Mg1^ix^—Mg7—Mg5^vi^	93.60 (4)
Mg8^xi^—Ru3—Mg6	68.69 (4)	Mg2—Mg7—Mg5^vi^	110.40 (5)
Mg5^iii^—Ru3—Mg6	109.70 (4)	Mg2^xi^—Mg7—Mg5^vi^	110.14 (5)
Mg5^xii^—Ru3—Mg6	59.53 (4)	Ru1^vi^—Mg7—Mg5^v^	82.50 (5)
Mg7—Ru3—Mg6	151.44 (3)	Ru1^v^—Mg7—Mg5^v^	54.16 (4)
Mg6^xi^—Ru3—Mg6	57.12 (5)	Ru3—Mg7—Mg5^v^	148.60 (3)
Mg1^ix^—Ru3—Mg2^xi^	96.80 (4)	Mg1^x^—Mg7—Mg5^v^	93.60 (4)
Mg1^x^—Ru3—Mg2^xi^	56.67 (4)	Mg1^ix^—Mg7—Mg5^v^	148.81 (6)
Mg8—Ru3—Mg2^xi^	160.18 (4)	Mg2—Mg7—Mg5^v^	110.14 (5)
Mg8^xi^—Ru3—Mg2^xi^	62.12 (4)	Mg2^xi^—Mg7—Mg5^v^	110.40 (5)
Mg5^iii^—Ru3—Mg2^xi^	129.33 (4)	Mg5^vi^—Mg7—Mg5^v^	62.81 (7)
Mg5^xii^—Ru3—Mg2^xi^	57.48 (4)	Ru1^vi^—Mg7—Mg1^v^	109.15 (5)
Mg7—Ru3—Mg2^xi^	60.19 (3)	Ru1^v^—Mg7—Mg1^v^	50.67 (3)
Mg6^xi^—Ru3—Mg2^xi^	123.05 (4)	Ru3—Mg7—Mg1^v^	111.39 (4)
Mg6—Ru3—Mg2^xi^	109.13 (4)	Mg1^x^—Mg7—Mg1^v^	102.14 (5)
Mg1^ix^—Ru3—Mg2	56.67 (4)	Mg1^ix^—Mg7—Mg1^v^	100.49 (3)
Mg1^x^—Ru3—Mg2	96.80 (4)	Mg2—Mg7—Mg1^v^	155.61 (4)
Mg8—Ru3—Mg2	62.12 (4)	Mg2^xi^—Mg7—Mg1^v^	52.07 (4)
Mg8^xi^—Ru3—Mg2	160.18 (4)	Mg5^vi^—Mg7—Mg1^v^	58.11 (4)
Mg5^iii^—Ru3—Mg2	57.48 (4)	Mg5^v^—Mg7—Mg1^v^	84.59 (5)
Mg5^xii^—Ru3—Mg2	129.33 (4)	Ru1^vi^—Mg7—Mg1^vi^	50.67 (3)
Mg7—Ru3—Mg2	60.19 (3)	Ru1^v^—Mg7—Mg1^vi^	109.15 (5)
Mg6^xi^—Ru3—Mg2	109.13 (4)	Ru3—Mg7—Mg1^vi^	111.39 (4)
Mg6—Ru3—Mg2	123.05 (4)	Mg1^x^—Mg7—Mg1^vi^	100.49 (3)
Mg2^xi^—Ru3—Mg2	120.39 (6)	Mg1^ix^—Mg7—Mg1^vi^	102.14 (5)
Ru1—Mg1—Ru3^ix^	133.24 (6)	Mg2—Mg7—Mg1^vi^	52.06 (4)
Ru1—Mg1—Mg2^ix^	158.02 (7)	Mg2^xi^—Mg7—Mg1^vi^	155.61 (4)
Ru3^ix^—Mg1—Mg2^ix^	68.72 (5)	Mg5^vi^—Mg7—Mg1^vi^	84.59 (5)
Ru1—Mg1—Mg2^ii^	56.50 (4)	Mg5^v^—Mg7—Mg1^vi^	58.11 (4)
Ru3^ix^—Mg1—Mg2^ii^	139.11 (6)	Mg1^v^—Mg7—Mg1^vi^	137.23 (8)
Mg2^ix^—Mg1—Mg2^ii^	109.04 (6)	Ru3—Mg8—Ru2^i^	160.19 (6)
Ru1—Mg1—Mg7^ix^	125.57 (5)	Ru3—Mg8—Mg3	104.72 (5)
Ru3^ix^—Mg1—Mg7^ix^	58.24 (4)	Ru2^i^—Mg8—Mg3	56.29 (3)
Mg2^ix^—Mg1—Mg7^ix^	62.40 (4)	Ru3—Mg8—Mg11^xiii^	132.15 (6)
Mg2^ii^—Mg1—Mg7^ix^	83.88 (6)	Ru2^i^—Mg8—Mg11^xiii^	57.89 (4)
Ru1—Mg1—Mg10	58.02 (4)	Mg3—Mg8—Mg11^xiii^	106.20 (5)
Ru3^ix^—Mg1—Mg10	90.63 (5)	Ru3—Mg8—Mg6^ix^	141.01 (6)
Mg2^ix^—Mg1—Mg10	131.50 (7)	Ru2^i^—Mg8—Mg6^ix^	57.18 (4)
Mg2^ii^—Mg1—Mg10	114.43 (6)	Mg3—Mg8—Mg6^ix^	104.40 (5)
Mg7^ix^—Mg1—Mg10	141.00 (6)	Mg11^xiii^—Mg8—Mg6^ix^	60.75 (5)
Ru1—Mg1—Mg8^ix^	125.08 (6)	Ru3—Mg8—Mg6	63.00 (4)
Ru3^ix^—Mg1—Mg8^ix^	56.24 (4)	Ru2^i^—Mg8—Mg6	122.68 (6)
Mg2^ix^—Mg1—Mg8^ix^	62.61 (5)	Mg3—Mg8—Mg6	102.34 (5)
Mg2^ii^—Mg1—Mg8^ix^	161.22 (7)	Mg11^xiii^—Mg8—Mg6	140.25 (6)
Mg7^ix^—Mg1—Mg8^ix^	104.61 (6)	Mg6^ix^—Mg8—Mg6	85.82 (5)
Mg10—Mg1—Mg8^ix^	69.38 (5)	Ru3—Mg8—Mg10	119.27 (6)
Ru1—Mg1—Ru1^xviii^	112.40 (5)	Ru2^i^—Mg8—Mg10	57.53 (4)
Ru3^ix^—Mg1—Ru1^xviii^	102.85 (5)	Mg3—Mg8—Mg10	56.49 (4)
Mg2^ix^—Mg1—Ru1^xviii^	53.62 (4)	Mg11^xiii^—Mg8—Mg10	108.17 (6)
Mg2^ii^—Mg1—Ru1^xviii^	56.34 (4)	Mg6^ix^—Mg8—Mg10	60.15 (5)
Mg7^ix^—Mg1—Ru1^xviii^	51.95 (4)	Mg6—Mg8—Mg10	66.51 (5)
Mg10—Mg1—Ru1^xviii^	166.21 (6)	Ru3—Mg8—Mg1^ix^	56.23 (4)
Mg8^ix^—Mg1—Ru1^xviii^	115.82 (5)	Ru2^i^—Mg8—Mg1^ix^	137.99 (6)
Ru1—Mg1—Mg4^xi^	93.92 (4)	Mg3—Mg8—Mg1^ix^	151.64 (6)
Ru3^ix^—Mg1—Mg4^xi^	132.67 (5)	Mg11^xiii^—Mg8—Mg1^ix^	80.13 (5)
Mg2^ix^—Mg1—Mg4^xi^	64.12 (5)	Mg6^ix^—Mg8—Mg1^ix^	102.72 (6)
Mg2^ii^—Mg1—Mg4^xi^	64.63 (4)	Mg6—Mg8—Mg1^ix^	87.73 (6)
Mg7^ix^—Mg1—Mg4^xi^	101.27 (5)	Mg10—Mg8—Mg1^ix^	148.90 (7)
Mg10—Mg1—Mg4^xi^	117.60 (5)	Ru3—Mg8—Mg2	65.25 (4)
Mg8^ix^—Mg1—Mg4^xi^	96.99 (5)	Ru2^i^—Mg8—Mg2	109.37 (6)
Ru1^xviii^—Mg1—Mg4^xi^	50.42 (2)	Mg3—Mg8—Mg2	99.43 (5)
Ru1—Mg1—Mg9	57.01 (3)	Mg11^xiii^—Mg8—Mg2	74.32 (5)
Ru3^ix^—Mg1—Mg9	135.87 (7)	Mg6^ix^—Mg8—Mg2	133.29 (7)
Mg2^ix^—Mg1—Mg9	108.60 (6)	Mg6—Mg8—Mg2	127.31 (6)
Mg2^ii^—Mg1—Mg9	84.62 (6)	Mg10—Mg8—Mg2	155.83 (7)
Mg7^ix^—Mg1—Mg9	161.78 (7)	Mg1^ix^—Mg8—Mg2	54.87 (5)
Mg10—Mg1—Mg9	57.12 (5)	Ru3—Mg8—Mg11	110.94 (5)
Mg8^ix^—Mg1—Mg9	82.68 (6)	Ru2^i^—Mg8—Mg11	56.10 (4)
Ru1^xviii^—Mg1—Mg9	109.83 (6)	Mg3—Mg8—Mg11	58.07 (4)
Mg4^xi^—Mg1—Mg9	60.91 (4)	Mg11^xiii^—Mg8—Mg11	59.92 (5)
Ru1—Mg1—Mg5^xiii^	79.76 (5)	Mg6^ix^—Mg8—Mg11	106.30 (6)
Ru3^ix^—Mg1—Mg5^xiii^	53.66 (4)	Mg6—Mg8—Mg11	158.63 (6)
Mg2^ix^—Mg1—Mg5^xiii^	122.19 (6)	Mg10—Mg8—Mg11	103.82 (6)
Mg2^ii^—Mg1—Mg5^xiii^	110.94 (6)	Mg1^ix^—Mg8—Mg11	106.07 (6)
Mg7^ix^—Mg1—Mg5^xiii^	82.33 (5)	Mg2—Mg8—Mg11	55.70 (5)
Mg10—Mg1—Mg5^xiii^	59.28 (5)	Ru3—Mg8—Mg5^iii^	55.21 (4)
Mg8^ix^—Mg1—Mg5^xiii^	87.04 (5)	Ru2^i^—Mg8—Mg5^iii^	105.56 (5)
Ru1^xviii^—Mg1—Mg5^xiii^	131.78 (6)	Mg3—Mg8—Mg5^iii^	57.45 (4)
Mg4^xi^—Mg1—Mg5^xiii^	173.67 (6)	Mg11^xiii^—Mg8—Mg5^iii^	117.95 (6)
Mg9—Mg1—Mg5^xiii^	115.06 (6)	Mg6^ix^—Mg8—Mg5^iii^	161.55 (7)
Ru1—Mg1—Mg7^i^	48.03 (2)	Mg6—Mg8—Mg5^iii^	100.60 (6)
Ru3^ix^—Mg1—Mg7^i^	99.98 (4)	Mg10—Mg8—Mg5^iii^	106.37 (6)
Mg2^ix^—Mg1—Mg7^i^	140.77 (6)	Mg1^ix^—Mg8—Mg5^iii^	94.85 (6)
Mg2^ii^—Mg1—Mg7^i^	54.61 (5)	Mg2—Mg8—Mg5^iii^	54.77 (5)
Mg7^ix^—Mg1—Mg7^i^	79.51 (3)	Mg11—Mg8—Mg5^iii^	62.71 (5)
Mg10—Mg1—Mg7^i^	84.15 (5)	Ru3—Mg8—Mg6^xi^	59.07 (4)
Mg8^ix^—Mg1—Mg7^i^	142.69 (7)	Ru2^i^—Mg8—Mg6^xi^	106.99 (5)
Ru1^xviii^—Mg1—Mg7^i^	96.16 (5)	Mg3—Mg8—Mg6^xi^	55.81 (4)
Mg4^xi^—Mg1—Mg7^i^	118.88 (5)	Mg11^xiii^—Mg8—Mg6^xi^	161.91 (6)
Mg9—Mg1—Mg7^i^	105.00 (4)	Mg6^ix^—Mg8—Mg6^xi^	121.84 (5)
Mg5^xiii^—Mg1—Mg7^i^	56.36 (4)	Mg6—Mg8—Mg6^xi^	54.63 (5)
Ru1^vi^—Mg2—Mg1^ix^	68.14 (5)	Mg10—Mg8—Mg6^xi^	65.12 (5)
Ru1^vi^—Mg2—Ru1^iii^	123.70 (6)	Mg1^ix^—Mg8—Mg6^xi^	114.59 (6)
Mg1^ix^—Mg2—Ru1^iii^	164.30 (7)	Mg2—Mg8—Mg6^xi^	104.81 (6)
Ru1^vi^—Mg2—Mg5^iii^	142.29 (7)	Mg11—Mg8—Mg6^xi^	104.20 (5)
Mg1^ix^—Mg2—Mg5^iii^	107.31 (7)	Mg5^iii^—Mg8—Mg6^xi^	53.08 (4)
Ru1^iii^—Mg2—Mg5^iii^	56.99 (4)	Ru1—Mg9—Ru1^xi^	134.47 (9)
Ru1^vi^—Mg2—Mg11	147.46 (7)	Ru1—Mg9—Mg10^xi^	157.83 (4)
Mg1^ix^—Mg2—Mg11	119.12 (7)	Ru1^xi^—Mg9—Mg10^xi^	58.38 (3)
Ru1^iii^—Mg2—Mg11	57.71 (4)	Ru1—Mg9—Mg10	58.38 (3)
Mg5^iii^—Mg2—Mg11	68.97 (5)	Ru1^xi^—Mg9—Mg10	157.83 (4)
Ru1^vi^—Mg2—Mg1^vi^	59.04 (4)	Mg10^xi^—Mg9—Mg10	117.45 (9)
Mg1^ix^—Mg2—Mg1^vi^	123.56 (6)	Ru1—Mg9—Mg11^xii^	135.50 (3)
Ru1^iii^—Mg2—Mg1^vi^	65.22 (4)	Ru1^xi^—Mg9—Mg11^xii^	57.61 (3)
Mg5^iii^—Mg2—Mg1^vi^	102.65 (6)	Mg10^xi^—Mg9—Mg11^xii^	65.30 (5)
Mg11—Mg2—Mg1^vi^	115.94 (6)	Mg10—Mg9—Mg11^xii^	100.34 (5)
Ru1^vi^—Mg2—Mg7	55.29 (4)	Ru1—Mg9—Mg11^iii^	57.61 (3)
Mg1^ix^—Mg2—Mg7	60.80 (5)	Ru1^xi^—Mg9—Mg11^iii^	135.51 (3)
Ru1^iii^—Mg2—Mg7	115.38 (5)	Mg10^xi^—Mg9—Mg11^iii^	100.34 (5)
Mg5^iii^—Mg2—Mg7	89.01 (6)	Mg10—Mg9—Mg11^iii^	65.30 (5)
Mg11—Mg2—Mg7	157.24 (8)	Mg11^xii^—Mg9—Mg11^iii^	153.45 (10)
Mg1^vi^—Mg2—Mg7	73.32 (5)	Ru1—Mg9—Mg1	55.36 (3)
Ru1^vi^—Mg2—Mg8	130.10 (6)	Ru1^xi^—Mg9—Mg1	109.88 (5)
Mg1^ix^—Mg2—Mg8	62.52 (5)	Mg10^xi^—Mg9—Mg1	144.55 (5)
Ru1^iii^—Mg2—Mg8	106.15 (5)	Mg10—Mg9—Mg1	59.45 (4)
Mg5^iii^—Mg2—Mg8	64.23 (5)	Mg11^xii^—Mg9—Mg1	80.16 (4)
Mg11—Mg2—Mg8	62.47 (5)	Mg11^iii^—Mg9—Mg1	107.84 (4)
Mg1^vi^—Mg2—Mg8	166.77 (7)	Ru1—Mg9—Mg1^xi^	109.88 (5)
Mg7—Mg2—Mg8	103.47 (6)	Ru1^xi^—Mg9—Mg1^xi^	55.36 (3)
Ru1^vi^—Mg2—Mg4^vi^	52.92 (3)	Mg10^xi^—Mg9—Mg1^xi^	59.45 (4)
Mg1^ix^—Mg2—Mg4^vi^	61.94 (4)	Mg10—Mg9—Mg1^xi^	144.55 (5)
Ru1^iii^—Mg2—Mg4^vi^	132.64 (6)	Mg11^xii^—Mg9—Mg1^xi^	107.84 (4)
Mg5^iii^—Mg2—Mg4^vi^	159.73 (6)	Mg11^iii^—Mg9—Mg1^xi^	80.16 (4)
Mg11—Mg2—Mg4^vi^	100.24 (6)	Mg1—Mg9—Mg1^xi^	145.67 (10)
Mg1^vi^—Mg2—Mg4^vi^	97.50 (5)	Ru1—Mg9—Mg4	52.20 (3)
Mg7—Mg2—Mg4^vi^	98.90 (4)	Ru1^xi^—Mg9—Mg4	91.88 (5)
Mg8—Mg2—Mg4^vi^	95.68 (5)	Mg10^xi^—Mg9—Mg4	118.45 (3)
Ru1^vi^—Mg2—Ru3	103.05 (5)	Mg10—Mg9—Mg4	107.56 (3)
Mg1^ix^—Mg2—Ru3	54.61 (4)	Mg11^xii^—Mg9—Mg4	143.32 (7)
Ru1^iii^—Mg2—Ru3	110.19 (5)	Mg11^iii^—Mg9—Mg4	62.75 (3)
Mg5^iii^—Mg2—Ru3	54.45 (4)	Mg1—Mg9—Mg4	93.80 (5)
Mg11—Mg2—Ru3	106.07 (6)	Mg1^xi^—Mg9—Mg4	59.43 (4)
Mg1^vi^—Mg2—Ru3	119.25 (6)	Ru1—Mg9—Mg4^xi^	91.88 (5)
Mg7—Mg2—Ru3	53.75 (5)	Ru1^xi^—Mg9—Mg4^xi^	52.20 (3)
Mg8—Mg2—Ru3	52.63 (4)	Mg10^xi^—Mg9—Mg4^xi^	107.56 (3)
Mg4^vi^—Mg2—Ru3	116.43 (5)	Mg10—Mg9—Mg4^xi^	118.45 (3)
Ru1^vi^—Mg2—Mg4^xiv^	92.82 (5)	Mg11^xii^—Mg9—Mg4^xi^	62.75 (3)
Mg1^ix^—Mg2—Mg4^xiv^	143.41 (7)	Mg11^iii^—Mg9—Mg4^xi^	143.32 (7)
Ru1^iii^—Mg2—Mg4^xiv^	51.22 (3)	Mg1—Mg9—Mg4^xi^	59.43 (4)
Mg5^iii^—Mg2—Mg4^xiv^	106.48 (5)	Mg1^xi^—Mg9—Mg4^xi^	93.80 (5)
Mg11—Mg2—Mg4^xiv^	62.15 (4)	Mg4—Mg9—Mg4^xi^	82.91 (6)
Mg1^vi^—Mg2—Mg4^xiv^	60.56 (4)	Ru1—Mg9—Mg6	118.97 (5)
Mg7—Mg2—Mg4^xiv^	133.38 (6)	Ru1^xi^—Mg9—Mg6	102.75 (4)
Mg8—Mg2—Mg4^xiv^	122.97 (6)	Mg10^xi^—Mg9—Mg6	62.88 (5)
Mg4^vi^—Mg2—Mg4^xiv^	81.57 (4)	Mg10—Mg9—Mg6	60.66 (5)
Ru3—Mg2—Mg4^xiv^	160.93 (5)	Mg11^xii^—Mg9—Mg6	54.81 (5)
Ru2^xix^—Mg3—Ru2^i^	180.000 (19)	Mg11^iii^—Mg9—Mg6	99.09 (7)
Ru2^xix^—Mg3—Mg10^iii^	61.03 (3)	Mg1—Mg9—Mg6	91.29 (5)
Ru2^i^—Mg3—Mg10^iii^	118.97 (3)	Mg1^xi^—Mg9—Mg6	121.00 (6)
Ru2^xix^—Mg3—Mg10	118.97 (3)	Mg4—Mg9—Mg6	161.83 (5)
Ru2^i^—Mg3—Mg10	61.03 (3)	Mg4^xi^—Mg9—Mg6	114.54 (3)
Mg10^iii^—Mg3—Mg10	180.0	Ru1—Mg9—Mg6^xi^	102.75 (4)
Ru2^xix^—Mg3—Mg6^xv^	121.09 (3)	Ru1^xi^—Mg9—Mg6^xi^	118.97 (5)
Ru2^i^—Mg3—Mg6^xv^	58.91 (3)	Mg10^xi^—Mg9—Mg6^xi^	60.66 (5)
Mg10^iii^—Mg3—Mg6^xv^	72.31 (4)	Mg10—Mg9—Mg6^xi^	62.88 (5)
Mg10—Mg3—Mg6^xv^	107.69 (4)	Mg11^xii^—Mg9—Mg6^xi^	99.09 (7)
Ru2^xix^—Mg3—Mg6^xi^	58.91 (3)	Mg11^iii^—Mg9—Mg6^xi^	54.81 (5)
Ru2^i^—Mg3—Mg6^xi^	121.09 (3)	Mg1—Mg9—Mg6^xi^	121.00 (6)
Mg10^iii^—Mg3—Mg6^xi^	107.69 (4)	Mg1^xi^—Mg9—Mg6^xi^	91.29 (5)
Mg10—Mg3—Mg6^xi^	72.31 (4)	Mg4—Mg9—Mg6^xi^	114.54 (3)
Mg6^xv^—Mg3—Mg6^xi^	180.0	Mg4^xi^—Mg9—Mg6^xi^	161.83 (5)
Ru2^xix^—Mg3—Mg11^iii^	59.25 (3)	Mg6—Mg9—Mg6^xi^	48.71 (6)
Ru2^i^—Mg3—Mg11^iii^	120.75 (3)	Ru1—Mg10—Mg3	102.11 (5)
Mg10^iii^—Mg3—Mg11^iii^	113.44 (4)	Ru1—Mg10—Ru2^i^	118.36 (5)
Mg10—Mg3—Mg11^iii^	66.56 (4)	Mg3—Mg10—Ru2^i^	58.03 (3)
Mg6^xv^—Mg3—Mg11^iii^	118.48 (4)	Ru1—Mg10—Mg9	59.03 (4)
Mg6^xi^—Mg3—Mg11^iii^	61.52 (4)	Mg3—Mg10—Mg9	110.34 (5)
Ru2^xix^—Mg3—Mg11	120.75 (3)	Ru2^i^—Mg10—Mg9	168.10 (6)
Ru2^i^—Mg3—Mg11	59.25 (3)	Ru1—Mg10—Mg5	56.69 (4)
Mg10^iii^—Mg3—Mg11	66.56 (4)	Mg3—Mg10—Mg5	61.53 (4)
Mg10—Mg3—Mg11	113.44 (4)	Ru2^i^—Mg10—Mg5	63.61 (4)
Mg6^xv^—Mg3—Mg11	61.52 (4)	Mg9—Mg10—Mg5	109.83 (6)
Mg6^xi^—Mg3—Mg11	118.48 (4)	Ru1—Mg10—Mg6^ix^	135.87 (6)
Mg11^iii^—Mg3—Mg11	180.0	Mg3—Mg10—Mg6^ix^	107.18 (5)
Ru2^xix^—Mg3—Mg8^iii^	60.01 (3)	Ru2^i^—Mg10—Mg6^ix^	57.21 (4)
Ru2^i^—Mg3—Mg8^iii^	119.99 (3)	Mg9—Mg10—Mg6^ix^	133.63 (6)
Mg10^iii^—Mg3—Mg8^iii^	62.81 (4)	Mg5—Mg10—Mg6^ix^	111.52 (6)
Mg10—Mg3—Mg8^iii^	117.19 (4)	Ru1—Mg10—Mg8	162.17 (6)
Mg6^xv^—Mg3—Mg8^iii^	67.90 (4)	Mg3—Mg10—Mg8	60.69 (4)
Mg6^xi^—Mg3—Mg8^iii^	112.10 (4)	Ru2^i^—Mg10—Mg8	58.40 (4)
Mg11^iii^—Mg3—Mg8^iii^	63.85 (4)	Mg9—Mg10—Mg8	119.96 (7)
Mg11—Mg3—Mg8^iii^	116.15 (4)	Mg5—Mg10—Mg8	112.81 (6)
Ru2^xix^—Mg3—Mg8	119.99 (3)	Mg6^ix^—Mg10—Mg8	59.49 (5)
Ru2^i^—Mg3—Mg8	60.01 (3)	Ru1—Mg10—Mg1	56.91 (4)
Mg10^iii^—Mg3—Mg8	117.19 (4)	Mg3—Mg10—Mg1	158.81 (6)
Mg10—Mg3—Mg8	62.81 (4)	Ru2^i^—Mg10—Mg1	126.25 (6)
Mg6^xv^—Mg3—Mg8	112.10 (4)	Mg9—Mg10—Mg1	63.43 (5)
Mg6^xi^—Mg3—Mg8	67.90 (4)	Mg5—Mg10—Mg1	100.34 (6)
Mg11^iii^—Mg3—Mg8	116.15 (4)	Mg6^ix^—Mg10—Mg1	89.18 (6)
Mg11—Mg3—Mg8	63.85 (4)	Mg8—Mg10—Mg1	140.47 (7)
Mg8^iii^—Mg3—Mg8	180.0	Ru1—Mg10—Mg5^xiii^	82.41 (5)
Ru2^xix^—Mg3—Mg5	115.39 (3)	Mg3—Mg10—Mg5^xiii^	112.50 (6)
Ru2^i^—Mg3—Mg5	64.61 (3)	Ru2^i^—Mg10—Mg5^xiii^	61.27 (4)
Mg10^iii^—Mg3—Mg5	118.43 (4)	Mg9—Mg10—Mg5^xiii^	126.94 (6)
Mg10—Mg3—Mg5	61.57 (4)	Mg5—Mg10—Mg5^xiii^	67.40 (6)
Mg6^xv^—Mg3—Mg5	58.87 (4)	Mg6^ix^—Mg10—Mg5^xiii^	56.21 (5)
Mg6^xi^—Mg3—Mg5	121.13 (4)	Mg8—Mg10—Mg5^xiii^	107.71 (6)
Mg11^iii^—Mg3—Mg5	67.55 (4)	Mg1—Mg10—Mg5^xiii^	65.22 (5)
Mg11—Mg3—Mg5	112.45 (4)	Ru1—Mg10—Mg11^iii^	55.04 (4)
Mg8^iii^—Mg3—Mg5	65.41 (4)	Mg3—Mg10—Mg11^iii^	58.67 (4)
Mg8—Mg3—Mg5	114.59 (4)	Ru2^i^—Mg10—Mg11^iii^	111.10 (6)
Ru2^xix^—Mg3—Mg5^iii^	64.61 (3)	Mg9—Mg10—Mg11^iii^	57.36 (4)
Ru2^i^—Mg3—Mg5^iii^	115.39 (3)	Mg5—Mg10—Mg11^iii^	64.73 (5)
Mg10^iii^—Mg3—Mg5^iii^	61.57 (4)	Mg6^ix^—Mg10—Mg11^iii^	165.75 (7)
Mg10—Mg3—Mg5^iii^	118.43 (4)	Mg8—Mg10—Mg11^iii^	108.27 (6)
Mg6^xv^—Mg3—Mg5^iii^	121.13 (4)	Mg1—Mg10—Mg11^iii^	104.95 (6)
Mg6^xi^—Mg3—Mg5^iii^	58.87 (4)	Mg5^xiii^—Mg10—Mg11^iii^	128.08 (6)
Mg11^iii^—Mg3—Mg5^iii^	112.45 (4)	Ru1—Mg10—Mg6	127.24 (6)
Mg11—Mg3—Mg5^iii^	67.55 (4)	Mg3—Mg10—Mg6	98.18 (5)
Mg8^iii^—Mg3—Mg5^iii^	114.59 (4)	Ru2^i^—Mg10—Mg6	113.76 (5)
Mg8—Mg3—Mg5^iii^	65.41 (4)	Mg9—Mg10—Mg6	68.30 (5)
Mg5—Mg3—Mg5^iii^	180.0	Mg5—Mg10—Mg6	158.34 (6)
Ru1^xvi^—Mg4—Ru1	180.0	Mg6^ix^—Mg10—Mg6	80.40 (5)
Ru1^xvi^—Mg4—Mg1^iv^	115.22 (3)	Mg8—Mg10—Mg6	56.50 (5)
Ru1—Mg4—Mg1^iv^	64.78 (3)	Mg1—Mg10—Mg6	97.77 (6)
Ru1^xvi^—Mg4—Mg1^xi^	64.78 (3)	Mg5^xiii^—Mg10—Mg6	131.98 (6)
Ru1—Mg4—Mg1^xi^	115.22 (3)	Mg11^iii^—Mg10—Mg6	99.14 (5)
Mg1^iv^—Mg4—Mg1^xi^	180.00 (6)	Ru1—Mg10—Mg6^xi^	106.00 (5)
Ru1^xvi^—Mg4—Mg9^xvi^	57.499 (8)	Mg3—Mg10—Mg6^xi^	55.38 (4)
Ru1—Mg4—Mg9^xvi^	122.501 (8)	Ru2^i^—Mg10—Mg6^xi^	104.77 (5)
Mg1^iv^—Mg4—Mg9^xvi^	59.67 (3)	Mg9—Mg10—Mg6^xi^	66.82 (5)
Mg1^xi^—Mg4—Mg9^xvi^	120.33 (3)	Mg5—Mg10—Mg6^xi^	107.50 (6)
Ru1^xvi^—Mg4—Mg9	122.501 (8)	Mg6^ix^—Mg10—Mg6^xi^	117.71 (5)
Ru1—Mg4—Mg9	57.499 (8)	Mg8—Mg10—Mg6^xi^	61.37 (5)
Mg1^iv^—Mg4—Mg9	120.33 (3)	Mg1—Mg10—Mg6^xi^	128.68 (6)
Mg1^xi^—Mg4—Mg9	59.67 (3)	Mg5^xiii^—Mg10—Mg6^xi^	166.04 (6)
Mg9^xvi^—Mg4—Mg9	180.0	Mg11^iii^—Mg10—Mg6^xi^	54.43 (4)
Ru1^xvi^—Mg4—Mg2^x^	54.33 (3)	Mg6—Mg10—Mg6^xi^	51.24 (5)
Ru1—Mg4—Mg2^x^	125.67 (3)	Ru1^iii^—Mg11—Ru2^i^	155.70 (6)
Mg1^iv^—Mg4—Mg2^x^	126.06 (4)	Ru1^iii^—Mg11—Mg2	60.28 (4)
Mg1^xi^—Mg4—Mg2^x^	53.94 (4)	Ru2^i^—Mg11—Mg2	116.17 (6)
Mg9^xvi^—Mg4—Mg2^x^	79.97 (3)	Ru1^iii^—Mg11—Mg9^iii^	59.37 (4)
Mg9—Mg4—Mg2^x^	100.03 (3)	Ru2^i^—Mg11—Mg9^iii^	128.41 (6)
Ru1^xvi^—Mg4—Mg2^ii^	125.67 (3)	Mg2—Mg11—Mg9^iii^	115.27 (7)
Ru1—Mg4—Mg2^ii^	54.33 (3)	Ru1^iii^—Mg11—Mg3	99.74 (4)
Mg1^iv^—Mg4—Mg2^ii^	53.94 (4)	Ru2^i^—Mg11—Mg3	56.62 (3)
Mg1^xi^—Mg4—Mg2^ii^	126.06 (4)	Mg2—Mg11—Mg3	104.09 (6)
Mg9^xvi^—Mg4—Mg2^ii^	100.03 (3)	Mg9^iii^—Mg11—Mg3	106.81 (6)
Mg9—Mg4—Mg2^ii^	79.97 (3)	Ru1^iii^—Mg11—Mg8^xiii^	142.98 (6)
Mg2^x^—Mg4—Mg2^ii^	180.00 (7)	Ru2^i^—Mg11—Mg8^xiii^	58.97 (4)
Ru1^xvi^—Mg4—Mg11^iii^	123.95 (3)	Mg2—Mg11—Mg8^xiii^	132.77 (7)
Ru1—Mg4—Mg11^iii^	56.05 (3)	Mg9^iii^—Mg11—Mg8^xiii^	88.30 (5)
Mg1^iv^—Mg4—Mg11^iii^	103.39 (4)	Mg3—Mg11—Mg8^xiii^	107.16 (5)
Mg1^xi^—Mg4—Mg11^iii^	76.61 (4)	Ru1^iii^—Mg11—Mg6^xv^	118.30 (5)
Mg9^xvi^—Mg4—Mg11^iii^	124.90 (4)	Ru2^i^—Mg11—Mg6^xv^	57.19 (4)
Mg9—Mg4—Mg11^iii^	55.10 (4)	Mg2—Mg11—Mg6^xv^	163.04 (7)
Mg2^x^—Mg4—Mg11^iii^	70.40 (4)	Mg9^iii^—Mg11—Mg6^xv^	72.30 (6)
Mg2^ii^—Mg4—Mg11^iii^	109.60 (4)	Mg3—Mg11—Mg6^xv^	58.98 (4)
Ru1^xvi^—Mg4—Mg11^xvii^	56.05 (3)	Mg8^xiii^—Mg11—Mg6^xv^	59.88 (5)
Ru1—Mg4—Mg11^xvii^	123.95 (3)	Ru1^iii^—Mg11—Mg11^xiii^	134.07 (4)
Mg1^iv^—Mg4—Mg11^xvii^	76.61 (4)	Ru2^i^—Mg11—Mg11^xiii^	57.31 (3)
Mg1^xi^—Mg4—Mg11^xvii^	103.39 (4)	Mg2—Mg11—Mg11^xiii^	76.07 (5)
Mg9^xvi^—Mg4—Mg11^xvii^	55.10 (4)	Mg9^iii^—Mg11—Mg11^xiii^	142.44 (7)
Mg9—Mg4—Mg11^xvii^	124.90 (4)	Mg3—Mg11—Mg11^xiii^	104.29 (4)
Mg2^x^—Mg4—Mg11^xvii^	109.60 (4)	Mg8^xiii^—Mg11—Mg11^xiii^	62.53 (5)
Mg2^ii^—Mg4—Mg11^xvii^	70.40 (4)	Mg6^xv^—Mg11—Mg11^xiii^	107.62 (5)
Mg11^iii^—Mg4—Mg11^xvii^	180.000 (14)	Ru1^iii^—Mg11—Mg8	107.59 (5)
Ru1^xvi^—Mg4—Mg2^iii^	122.97 (3)	Ru2^i^—Mg11—Mg8	57.11 (4)
Ru1—Mg4—Mg2^iii^	57.03 (3)	Mg2—Mg11—Mg8	61.83 (5)
Mg1^iv^—Mg4—Mg2^iii^	54.81 (4)	Mg9^iii^—Mg11—Mg8	159.99 (6)
Mg1^xi^—Mg4—Mg2^iii^	125.19 (4)	Mg3—Mg11—Mg8	58.08 (4)
Mg9^xvi^—Mg4—Mg2^iii^	79.12 (3)	Mg8^xiii^—Mg11—Mg8	108.10 (6)
Mg9—Mg4—Mg2^iii^	100.88 (3)	Mg6^xv^—Mg11—Mg8	105.55 (6)
Mg2^x^—Mg4—Mg2^iii^	85.53 (5)	Mg11^xiii^—Mg11—Mg8	57.55 (5)
Mg2^ii^—Mg4—Mg2^iii^	94.47 (5)	Ru1^iii^—Mg11—Mg10^iii^	56.04 (4)
Mg11^iii^—Mg4—Mg2^iii^	53.84 (4)	Ru2^i^—Mg11—Mg10^iii^	106.11 (5)
Mg11^xvii^—Mg4—Mg2^iii^	126.16 (4)	Mg2—Mg11—Mg10^iii^	103.72 (6)
Ru1^xvi^—Mg4—Mg2^xvii^	57.03 (3)	Mg9^iii^—Mg11—Mg10^iii^	57.34 (4)
Ru1—Mg4—Mg2^xvii^	122.97 (3)	Mg3—Mg11—Mg10^iii^	54.77 (4)
Mg1^iv^—Mg4—Mg2^xvii^	125.19 (4)	Mg8^xiii^—Mg11—Mg10^iii^	123.16 (6)
Mg1^xi^—Mg4—Mg2^xvii^	54.81 (4)	Mg6^xv^—Mg11—Mg10^iii^	66.65 (5)
Mg9^xvi^—Mg4—Mg2^xvii^	100.88 (3)	Mg11^xiii^—Mg11—Mg10^iii^	158.75 (6)
Mg9—Mg4—Mg2^xvii^	79.12 (3)	Mg8—Mg11—Mg10^iii^	103.13 (6)
Mg2^x^—Mg4—Mg2^xvii^	94.47 (5)	Ru1^iii^—Mg11—Mg4^xiv^	52.36 (2)
Mg2^ii^—Mg4—Mg2^xvii^	85.53 (5)	Ru2^i^—Mg11—Mg4^xiv^	150.92 (5)
Mg11^iii^—Mg4—Mg2^xvii^	126.16 (4)	Mg2—Mg11—Mg4^xiv^	64.02 (4)
Mg11^xvii^—Mg4—Mg2^xvii^	53.84 (4)	Mg9^iii^—Mg11—Mg4^xiv^	62.14 (5)
Mg2^iii^—Mg4—Mg2^xvii^	180.00 (6)	Mg3—Mg11—Mg4^xiv^	152.10 (5)
Ru1—Mg5—Ru3^iii^	127.09 (6)	Mg8^xiii^—Mg11—Mg4^xiv^	98.36 (5)
Ru1—Mg5—Mg6^xv^	160.41 (7)	Mg6^xv^—Mg11—Mg4^xiv^	130.20 (6)
Ru3^iii^—Mg5—Mg6^xv^	64.20 (4)	Mg11^xiii^—Mg11—Mg4^xiv^	97.28 (3)
Ru1—Mg5—Mg2^iii^	60.80 (4)	Mg8—Mg11—Mg4^xiv^	124.15 (5)
Ru3^iii^—Mg5—Mg2^iii^	68.06 (5)	Mg10^iii^—Mg11—Mg4^xiv^	101.66 (5)
Mg6^xv^—Mg5—Mg2^iii^	121.82 (7)	Ru1^iii^—Mg11—Mg5^iii^	53.03 (4)
Ru1—Mg5—Mg3	100.33 (5)	Ru2^i^—Mg11—Mg5^iii^	103.98 (5)
Ru3^iii^—Mg5—Mg3	104.28 (5)	Mg2—Mg11—Mg5^iii^	55.38 (5)
Mg6^xv^—Mg5—Mg3	60.11 (4)	Mg9^iii^—Mg11—Mg5^iii^	101.57 (5)
Mg2^iii^—Mg5—Mg3	104.07 (6)	Mg3—Mg11—Mg5^iii^	56.38 (4)
Ru1—Mg5—Mg10	58.99 (4)	Mg8^xiii^—Mg11—Mg5^iii^	162.57 (6)
Ru3^iii^—Mg5—Mg10	160.57 (6)	Mg6^xv^—Mg11—Mg5^iii^	109.19 (6)
Mg6^xv^—Mg5—Mg10	104.98 (6)	Mg11^xiii^—Mg11—Mg5^iii^	113.06 (7)
Mg2^iii^—Mg5—Mg10	109.38 (6)	Mg8—Mg11—Mg5^iii^	59.74 (4)
Mg3—Mg5—Mg10	56.90 (4)	Mg10^iii^—Mg11—Mg5^iii^	54.59 (5)
Ru1—Mg5—Ru2^i^	113.22 (5)	Mg4^xiv^—Mg11—Mg5^iii^	98.95 (4)

Symmetry codes: (i) *x*+1/2, *y*−1/2, *z*; (ii) −*x*+1/2, *y*−1/2, −*z*+1/2; (iii) −*x*+1/2, −*y*+1/2, −*z*; (iv) *x*, −*y*, *z*−1/2; (v) *x*−1/2, *y*+1/2, *z*; (vi) −*x*+1/2, *y*+1/2, −*z*+1/2; (vii) *x*, −*y*+1, *z*−1/2; (viii) −*x*, −*y*+1, −*z*+1; (ix) −*x*+1/2, −*y*+1/2, −*z*+1; (x) *x*−1/2, −*y*+1/2, *z*−1/2; (xi) −*x*, *y*, −*z*+1/2; (xii) *x*−1/2, −*y*+1/2, *z*+1/2; (xiii) −*x*+1, *y*, −*z*+1/2; (xiv) *x*+1/2, *y*+1/2, *z*; (xv) *x*+1/2, −*y*+1/2, *z*−1/2; (xvi) −*x*, −*y*, −*z*; (xvii) *x*−1/2, *y*−1/2, *z*; (xviii) *x*, −*y*, *z*+1/2; (xix) −*x*, −*y*+1, −*z*.

## References

[bb1] Brandenburg, K. & Putz, H. (2017). *DIAMOND*. Crystal Impact GbR, Bonn, Germany.

[bb2] Bruker (2015). *APEX3* and *SAINT*. Bruker AXS Inc. Madison, Wisconsin, USA, 2008.

[bb3] Hlukhyy, V. & Pöttgen, R. (2005). *J. Solid State Chem.* **178**, 79–84.

[bb4] Krause, L., Herbst-Irmer, R., Sheldrick, G. M. & Stalke, D. (2015). *J. Appl. Cryst.* **48**, 3–10. 10.1107/S1600576714022985 PMC4453166 26089746

[bb5] Liu, C. & Fan, C. (2018). *IUCrData*, **3**, x180363.

[bb6] Miyazaki, Y., Okada, J. T. & Kimura, K. (2007). *Philos. Mag.* **87**, 2701–2706.

[bb7] Pöttgen, R., Hlukhyy, V., Baranov, A. & Grin, Y. (2008). *Inorg. Chem.* **47**, 6051–6055. 10.1021/ic800387a 18549193

[bb8] Schweitzer, K. & Jung, W. (1985). *Z. Anorg. Allge Chem.* **530**, 127–134.

[bb9] Sheldrick, G. M. (2015*a*). *Acta Cryst.* A**71**, 3–8.

[bb10] Sheldrick, G. M. (2015*b*). *Acta Cryst.* C**71**, 3–8.

[bb11] Westin, L. & Edshammar, L. E. (1973). *Chem. Scr.* **3**, 15–22.

[bb12] Westrip, S. P. (2010). *J. Appl. Cryst.* **43**, 920–925.

